# Integrated Microfluidics for Single‐Cell Separation and On‐Chip Analysis: Novel Applications and Recent Advances

**DOI:** 10.1002/smsc.202300206

**Published:** 2024-02-02

**Authors:** Hazal Kutluk, Martina Viefhues, Iordania Constantinou

**Affiliations:** ^1^ Institute of Microtechnology (IMT) Technische Universität Braunschweig Alte Salzdahlumer Str. 203 38124 Braunschweig Germany; ^2^ Center of Pharmaceutical Engineering (PVZ) Technische Universität Braunschweig Franz‐Liszt‐Str. 35a 38106 Braunschweig Germany; ^3^ Experimental Biophysics & Applied Nanoscience Faculty of Physics Bielefeld University Universitätsstrasse 25 33615 Bielefeld Germany

**Keywords:** integrated microfluidics, single‐cell analyses, single‐cell isolations, single‐cell sortings

## Abstract

From deciphering infection and disease mechanisms to identifying novel biomarkers and personalizing treatments, the characteristics of individual cells can provide significant insights into a variety of biological processes and facilitate decision‐making in biomedical environments. Conventional single‐cell analysis methods are limited in terms of cost, contamination risks, sample volumes, analysis times, throughput, sensitivity, and selectivity. Although microfluidic approaches have been suggested as a low‐cost, information‐rich, and high‐throughput alternative to conventional single‐cell isolation and analysis methods, limitations such as necessary off‐chip sample pre‐ and post‐processing as well as systems designed for individual workflows have restricted their applications. In this review, a comprehensive overview of recent advances in integrated microfluidics for single‐cell isolation and on‐chip analysis in three prominent application domains are provided: investigation of somatic cells (particularly cancer and immune cells), stem cells, and microorganisms. Also, the use of conventional cell separation methods (e.g., dielectrophoresis) in unconventional or novel ways, which can advance the integration of multiple workflows in microfluidic systems, is discussed. Finally, a critical discussion related to current limitations of integrated microfluidic single‐cell workflows and how they could be overcome is provided.

## Graphical abstract

Fully integrated microsystems have the potential to revolutionize single‐cell analysis by upholding common advantages of microfluidics while eliminating the need for off‐chip sample processing. In this manuscript, multiple emerging examples capable of isolation and analysis of different types of single cells are discussed. In these systems, content‐rich information is provided through the integration of various workflows on a single platform.

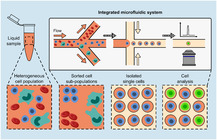

## 1 Introduction

Biological and biochemical analyses at the single‐cell level have proven to be pivotal in various branches of biology and medicine, as they reveal heterogeneities in cellular populations and provide valuable insights into the mechanisms and pathways involved in (patho)physiological processes.^[^ ^1^, ^2^ ^]^ In both research and clinical settings, single‐cell analysis has a wide range of applications, including drug discovery and screening,^[^ ^2^, ^3^ ^]^ investigation of host–pathogen interactions,^[^ ^4^, ^5^ ^]^ the study of cell development,^[^ ^6^ ^]^ and disease diagnosis and treatment prognosis, particularly when a low number of cells are available.^[^ ^7^, ^8^, ^9^ ^]^ The majority of single‐cell workflows begin with the sorting of seemingly homogenous cell subpopulations from a sample containing a heterogenous mixture of cells, followed by the isolation and analysis of individual cells. In conventional clinical and laboratory settings, this series of processes is often laborious, and limited by the cost and/or throughput required to characterize large cell populations and the difficulties associated with analyzing small sample volumes.

**Figure 1:**
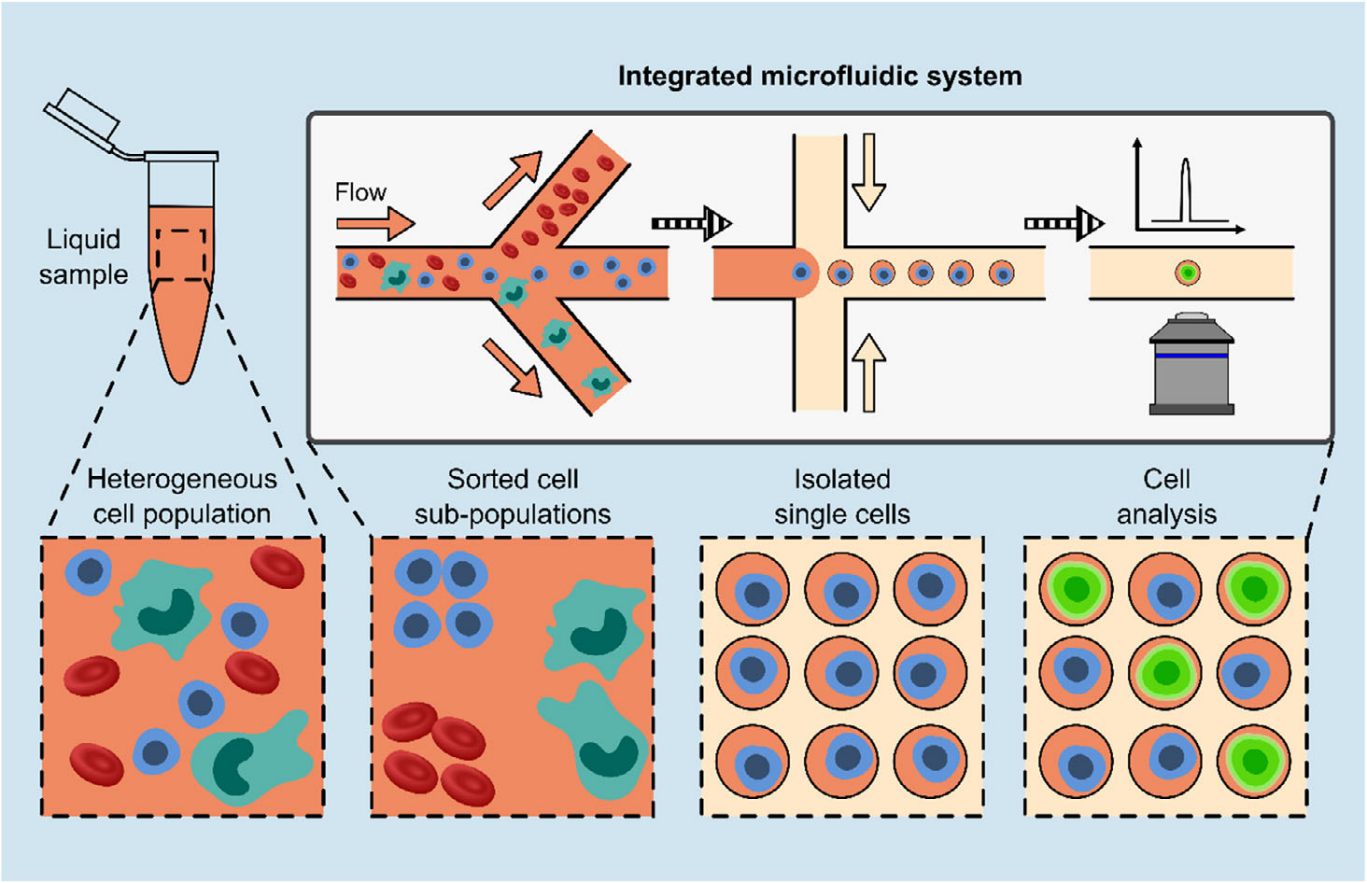
Example of a fully integrated microfluidic system for on‐chip cell sorting, single‐cell isolation, and analysis in flow. Illustrations are not to scale. Initially, a liquid sample containing a heterogeneous cell population, such as blood, is sorted to obtain cellular subpopulations (here steered toward three different channels). Single cells belonging to the subpopulation of interest are isolated (here via droplet encapsulation). Finally, isolated single cells are analyzed on‐chip (here optically via fluorescence imaging) to reveal individual cellular properties. Although in this example illustration cell sorting, isolation, and analysis take place in flow, in‐flow processing is not a general requirement for all integrated microfluidic systems.

Microfluidic single‐cell analysis systems arose as a promising way to overcome current limitations of conventional single‐cell workflows. Due to the versatility in design and cell‐relevant length scales of microchannels, microfluidic systems provide an ideal environment for precise cell manipulation and single‐cell analysis. In addition, compared to traditional cell‐based assays commonly performed in petri dishes, microtiter plates, and flasks, microfluidics allow higher throughputs and therefore increased biological data relevance.^[^ ^10^ ^]^ Despite the potential, single‐cell analysis using microfluidics still heavily relies on off‐chip sample preparation and/or handling, as most microfluidic systems are designed to facilitate a single experimental process step as opposed to entire workflows. Such single‐step microfluidic systems have been reviewed countless times and do not belong to the scope of this work.^[^ ^11^, ^12^, ^13^ ^]^ Herein, we use the term “integrated microfluidics” to describe multifunctional microfluidic systems that can perform multiple on‐chip single‐cell operations with no or minimal off‐chip sample handling between individual process steps (**Figure** **1**). While the establishment of complete workflows into a single microfluidic platform is a challenging task that demands extensive design considerations and the integration of multiple technologies, recent progress in this field has been promising.

In this review, we present an overview of emerging microfluidic platforms that demonstrate significant advances toward the integration of complete single‐cell workflows on‐chip, while particularly focusing on systems with integrated on‐chip analysis. In the first part, we introduce the prominent microfluidic methods traditionally used for cell sorting (i.e., sorting of heterogeneous cell populations into homogeneous subpopulations of interest) and single‐cell isolation, as well as the prevalent methods used for on‐chip single‐cell analysis. We also explore, where applicable, particular applications that utilize traditional cell separation and analysis methods in unconventional/novel ways that can be used to advance the integration of multiple workflows in microfluidic systems. Most other published reviews discussing single‐cell isolation and analysis methods maintain a narrow focus, for example, applications of such methods in omics for drug development.^[^ ^14^, ^15^ ^]^ Similarly, reviews of integrated platforms tend to focus on the isolation and analysis of specific cell types, e.g., microbial cells.^[^ ^16^ ^]^ Herein, we take a more comprehensive approach and attempt to introduce specialist and nonspecialist readers to the most commonly used isolation and analysis methods and the application of integrated platforms for the characterization of a variety of single‐cell types for a variety of applications. Specifically, in the second part of the review, we present recent advances in integrated microfluidics and their application for the analysis of somatic cells (particularly cancer and immune cells), as well as stem cells and microorganisms. Finally, we critically discuss the challenges that hinder the development and widespread use of integrated microfluidics for single‐cell analysis and identify avenues that could propel such technologies forward.

## 2 Methods for Cell Sorting and Isolation in Integrated Microfluidics

Cell separation is a common process starting point in cell biology and biotechnology laboratories used for sample preparation. Cells are sorted/isolated for molecular analysis, clonal colony generation, and specific cell incorporation into functional assays. In all cases, it is critical that cells are healthy, verifiable, and phenotypically characterized with high accuracy.^[^ ^12^, ^17^, ^18^ ^]^ Conventional cell separation methods include centrifugation gradients, magnetic‐activated cell sorting, fluorescence‐activated cell sorting, and manual cell picking.^[^ ^19^ ^]^ These techniques offer a range of resolutions, throughputs, and cost. Microfluidic‐based cell separation approaches offer many advantages over conventional techniques including the use of small sample and reagent volumes, portability, low costs, and improved sterility.^[^ ^10^ ^]^

Methods used for cell sorting and isolation in (integrated) microfluidics are broadly classified as active or passive. Active cell separation relies on the interaction between cells traveling through microfluidic channels and external force fields applied across the channels that cause a change in their position. Prominent active cell separation methods we discuss later include dielectrophoresis (DEP), magnetophoresis, acoustophoresis, and optical tweezers. The fundamental equations governing the physical principles behind these active separation methods are summarized in Table S1 (see Supporting Information). Passive systems primarily make use of hydrodynamic and inertial forces for cell separation, as well as geometric structures. In this case, cell manipulation depends on inherent property differences between cells, such as their size, shape, or density. In addition to active cell separation methods, later we also discuss passive separation methods such as inertial and hydrodynamic focusing, microarchitecture‐based separation (micropillars and deterministic lateral displacement, microwells, microchambers, and microtraps), and droplet‐based microfluidics. At the end of Section 2, **Table** **1** is used to summarize and compare the active and passive separation methods discussed later, outlining their respective advantages and limitations. Furthermore, Table 1 summarizes the discussed innovative applications, which could potentially facilitate novel approaches in integrated microfluidic systems.

**Table 1:** Advantages and drawbacks of the prominent cell separation methods discussed in this manuscript. Novel approaches/methods developed for enhanced applications are summarized as well

Active cell separation method	Advantages	Drawbacks	Novel applications
Dielectrophoresis	• Label‐free, contact‐free separation • Separation based on intrinsic dielectric polarizability^[^ ^21^ ^]^ • Unique electrical characteristics of different cell types identifiable^[^ ^21^ ^]^ • Easy integration as microelectrode integration into microfluidic systems well‐established • Wireless operation possible via bipolar electrode architecture^[^ ^42^, ^132^, ^252^, ^253^ ^]^	• DEP force acting on a cell/particle significantly varied as a function of distance away from an electrode edge^[^ ^21^ ^]^ • Adverse effects on samples due to Joule heating when high electric field strength is required^[^ ^25^ ^]^ • Possible degradation of the electrode and the nearby biological samples due to the electrochemical activity at the electrode‐buffer interface^[^ ^25^, ^26^, ^153^ ^]^ • Dependence on the electrical properties of the medium, which may necessitate specific buffer conditions that are optimal for cells^[^ ^26^, ^28^, ^42^, ^201^ ^]^	• Besides trapping, controlled transport and motion of cells and particles based on DEP^[^ ^42^, ^43^ ^]^ • Use of cheaper materials and easier fabrication methods^[^ ^238^, ^239^, ^250^ ^]^ • Combination with other separation methods^[^ ^251^ ^]^
Magnetophoresis	• Well‐established magnetic cell labeling^[^ ^21^ ^]^ • Magnetic particle kits commercially available • Magnetic particle properties do not degrade and are commonly not affected by chemistry^[^ ^21^ ^]^ • No significant magnetic noise usually present to interfere with cell/particle manipulation^[^ ^21^ ^]^ • Relatively little heat generation^[^ ^53^ ^]^ • Magnetic fields can penetrate most microfluidic materials^[^ ^53^ ^]^	• Most often label‐based • Biocompatibility of some magnetic particles not thoroughly studied^[^ ^201^ ^]^ • Biocompatibility of ferrofluids for label‐free magnetophoresis a key challenge^[^ ^46^ ^]^ • Generation of controlled magnetic forces not straightforward^[^ ^21^ ^]^ • Sorting efficiency limited by the strength of the magnetic field and the magnetic properties of the medium and particles	• Micro‐patterning of magnetic micro‐sources to establish localized, high magnetic fields^[^ ^54^, ^55^, ^56^, ^57^ ^]^ • Magnetic microtweezers for single cell manipulation^[^ ^57^ ^]^ • Negative selection of non‐targeted cells^[^ ^60^ ^]^ • Magnetomicrofluidic circuits for spatial single cell control^[^ ^254^, ^255^ ^]^ • Use of alternating magnetic fields for advanced particle movement^[^ ^62^ ^]^
Acoustophoresis	• Label‐free, contact‐free separation^[^ ^63^ ^]^ • Large operation/penetration distances^[^ ^21^ ^]^ • Large volume and high‐throughput processing^[^ ^63^ ^]^ • Application independent of properties like pH, ionic strength, or charge^[^ ^64^ ^]^	• Bulk piezoelectric transducers pose geometric limitations to integration^[^ ^68^ ^]^ • Less sensitive sample discrimination than on dielectric polarizability^[^ ^21^ ^]^ • Cell manipulation efficiency influenced by the properties of the medium and the microfluidic channel geometry^[^ ^65^, ^66^ ^]^ • Requires the use materials with high specific acoustic impedances relative to the fluid^[^ ^70^ ^]^ • Most effective for the manipulation of spherical cells^[^ ^75^ ^]^	• Applications of acoustophoretic principles in polymer‐based platforms.^[^ ^66^, ^70^, ^71^, ^72^ ^]^ • Size‐independent cell separation via isoacoustic focusing^[^ ^75^ ^]^ • Generation of 2D acoustic standing waves in microchannels to focus non‐spherical cells^[^ ^76^ ^]^ • Acoustic cell washing^[^ ^77^, ^78^ ^]^ • Use of secondary acoustic radiation forces or acoustic streaming to manipulate single‐cell motion^[^ ^81^, ^82^, ^84^ ^]^
Optical Tweezers	• Label‐free, contact‐free separation^[^ ^86^, ^95^, ^256^ ^]^ • High force resolution^[^ ^86^ ^]^ • Application possible both in flow and static conditions^[^ ^91^, ^256^ ^]^ • Photo, thermal and mechanical influence on cells might be utilized for cellular and molecular analyses (e.g., light scalpels, intracellular deliveries, membrane fusion)^[^ ^259^ ^]^	• Handling often limited to small, single cells, therefore low throughput and scalability^[^ ^90^, ^91^, ^257^ ^]^ • Limited speed of the manipulated particles^[^ ^257^ ^]^ • Sophisticated and expensive equipment^[^ ^90^, ^91^, ^95^ ^]^ • Large power needed to create reasonable trapping forces^[^ ^91^ ^]^ • Potential for photo, thermal and mechanical damage to cells^[^ ^90^, ^91^, ^259^ ^]^ • Trapping in crowded environments challenging^[^ ^257^, ^260^ ^]^	• 3D trapping via optical setups that enhance the stiffness of optical traps^[^ ^89^ ^]^ • Miniaturization of optical elements and their integration into microfluidic systems^[^ ^91^, ^94^, ^95^, ^258^ ^]^ • Optoelectronic tweezers^[^ ^90^ ^]^

Passive cell separation method	Advantages	Drawbacks	Novel applications
Inertial and hydrodynamic focusing	• Label‐free, contact‐free separation • Continuous sample processing possible^[^ ^98^ ^]^ • Simple fabrication process that only relies on channel geometry	• Dilution of crucial biological elements, such as cell secretion molecules, due to continuous perfusion^[^ ^201^ ^]^ • Clogging risk due to high cell densities^[^ ^106^ ^]^ • Complex flow patterns need careful design^[^ ^98^ ^]^ • Limitations in sorting cells with similar size and density • Limited control of cell positions in the vertical dimension of the channel^[^ ^103^, ^104^ ^]^	• 3D flow focusing for enhanced positioning of cells in horizontal and vertical dimensions of the channel^[^ ^103^, ^104^ ^]^
Micropillars and DLD	• Label‐free separation^[^ ^261^ ^]^ • Continuous sample processing possible^[^ ^261^, ^262^ ^]^ • Large size separation range (nanometer‐ to millimeter‐sized objects)^[^ ^110^, ^262^ ^]^	• Separation principles not completely understood^[^ ^262^ ^]^ • Sorting efficiency highly dependent on the geometry and precise dimensions of the pillar array^[^ ^109^, ^262^ ^]^	• Combination with DEP for enhanced separation efficiency^[^ ^192^ ^]^
Microwells, microchambers and microtraps	• Label‐free separation • High versatility and compatibility with miniaturized bioassays for enhanced separation and analysis capabilities^[^ ^202^ ^]^ • Well‐controlled cell positions allow long‐term single‐cell analysis • Facilitates single‐cell‐bead pairings and single‐cell co‐cultures^[^ ^195^, ^203^ ^]^	• Motile cells might leave the compartments^[^ ^263^ ^]^ • Limited control in ratios and distances between cells in single‐cell co‐culture^[^ ^202^ ^]^ • Potential cross‐talk between wells or traps^[^ ^127^ ^]^ • Targeted delivery challenging, as reagents are distributed throughout the array^[^ ^127^ ^]^ • Limited dynamic range in sorting cells of different sizes or types	• Stackable microwells for controlled single‐cell co‐cultures^[^ ^115^, ^116^ ^]^ • Combination with microvalves to prevent‐cross‐talk between chambers and enable targeted solution exchange^[^ ^195^ ^]^
Droplet‐based microfluidics	• Label‐free, contact‐free separation • Controlled confinement of single cells in well‐defined microenvironments^[^ ^125^, ^127^, ^201^ ^]^ • No cross‐talk between droplets^[^ ^125^, ^127^, ^201^ ^]^ • Minimal sample and reagent usage, therefore cost reduction^[^ ^125^, ^127^ ^]^ • Suitable for a variety of assays, including genomic, transcriptomic, proteomic, and metabolic studies^[^ ^125^ ^]^ • Fast reaction times and real‐time detection^[^ ^127^, ^131^ ^]^ • High‐throughput^[^ ^126^, ^127^ ^]^	• Cell encapsulation into droplets non‐selective and based on Poisson statistics, which might lead to a high number of empty droplets^[^ ^131^, ^132^, ^189^ ^]^ • Droplet size crucial in cell viability, nutrient replenishment and waste removal in droplets are limited^[^ ^136^, ^201^ ^]^ • Culturing of single adherent cells challenging^[^ ^136^, ^201^, ^264^ ^]^ • Potential for droplet coalescence or instability, affecting assay results^[^ ^265^, ^266^ ^]^	• Application of image recognition techniques for selective droplet manipulation^[^ ^131^ ^]^ • In‐droplet solution exchange for nutrient supply and waste removal^[^ ^133^, ^134^ ^]^ • Gentle manipulation of large droplets without breakage for improved cell viability for longer periods^[^ ^135^ ^]^ • Cell attachment on microgels prior to droplet encapsulation to establish adherent cell cultures in droplets^[^ ^140^ ^]^ • Precise positioning and targeted manipulation of droplets leveraging microwells, magnetophoresis, DMF or microvalves^[^ ^124^, ^141^, ^142^, ^143^, ^204^ ^]^

Cell separation methods can be additionally classified as affinity‐based and non‐affinity based.^[^ ^20^ ^]^ Affinity‐based methods require either the functionalization of microchannel surfaces with ligands that target the proteins on the cell surface, such as antibodies, or the labeling of cells with specific markers, such as aptamers or antibody‐conjugated particles. Separation is realized via the immobilization of the (labeled) target cells on a capture surface in a microchannel with modified biochemical properties (passive separation), or via the application of external force fields (active separation, e.g., in the case of magnetophoresis discussed in Section 2.1.2).

### 2.1 Active Cell Sorting and Isolation Methods—Introduction of Methods and Novel Applications

#### 2.1.1 DEP

DEP refers to the motion of electrically polarizable objects in nonuniform electric fields.^[^ ^21^ ^]^ Although electrically neutral, cells can become polarized (i.e., form electric dipoles) when placed in electric fields and can therefore be manipulated using DEP.^[^ ^22^ ^]^ In microfluidics, DEP is arguably the most commonly utilized active separation method, finding countless applications in sorting cells, e.g., based on cell type or at different parts in their life cycle.^[^ ^23^, ^24^, ^25^, ^26^ ^]^ Important advantages of DEP include the lack of labels and the well‐established protocols outlining electrode integration into microsystems.^[^ ^21^ ^]^

The application of DEP for well‐controlled cell separation requires precise control over the velocity and the direction of dielectrophoretic cell migration. The velocity of cell migration depends on the magnitude of the dielectrophoretic force exerted on a cell, which is a function of the cell size and the dielectric properties of both the cell and the surrounding medium.^[^ ^27^ ^]^ The direction of cell migration is a function of the polarizability of the cell relative to that of the medium it is suspended in.^[^ ^28^ ^]^ When the polarizability of the cell is greater than that of the medium, the cell will move in the direction of the field gradient experiencing a positive dielectrophoretic force (positive DEP). Negative DEP occurs when the cell is less polarizable than the surrounding medium and it forces the cell toward areas of low field strength.^[^ ^21^ ^]^ One significant disadvantage of DEP is the weakening of the dielectrophoretic force in media with high electrolyte concentrations, such as physiological salt solutions, due to the diminishing contrast in polarizability between the cells and the medium. Hence, in such physiological media, the control of migration velocity and direction requires careful execution.

The nonuniform electric fields essential for dielectrophoretic applications are commonly generated using integrated microelectrodes or insulating features structured in microchannels to reduce the cross‐sectional area and increase the electric field strength.^[^ ^25^, ^26^, ^28^ ^]^ As the dielectrophoretic force needed for the movement of cells and particles is proportional to the gradient of the squared electric field strength (see Table S1, Supporting Information), high electric field strengths are commonly desired. Yet, for applications requiring the use of high electric fields, Joule (or Ohmic) heating has to be considered as a potential limitation. Joule heating refers to the thermal energy generated when an electric current passes through a conductor, due to the electrical resistance it encounters.^[^ ^29^, ^30^ ^]^ This phenomenon can cause a temperature increase of the sample and/or the buffer, which might lead to unintended changes on the analytical process or the sample itself.^[^ ^25^ ^]^ Moreover, temperature gradients act as body forces on the surrounding liquid generating an electrothermal fluid flow,^[^ ^31^ ^]^ which reduces the control on sample and fluid migration. In cases where cell viability and function are less sensitive to changes in media, the effects of Joule heating can be reduced by adjusting the conductivity of the solution/media as the local power generated by Joule heating is proportional to the conductivity of the electrolyte solution.^[^ ^26^, ^32^ ^]^

Over the years, DEP‐based separation has been integrated into microfluidic systems for countless applications.^[^ ^25^ ^]^ The versatility of the technology combined with advancements in related microtechnology has fueled various investigations including the optimization of insulating structures (e.g., constriction channels)^[^ ^33^, ^34^, ^35^, ^36^, ^37^, ^38^ ^]^ and electrode geometries (e.g., tilted electrodes, electrodes on multiple faces of the microfluidic channel).^[^ ^39^, ^40^, ^41^ ^]^ Novel applications of DEP stem from these investigations and their reimagination in new settings. For example, Wu et al. developed a microfluidic device that utilizes DEP to not only separate and trap single yeast cells, but also to allow for their controlled motion in a noninvasive manner both along their rotational and translational axes.^[^ ^42^ ^]^ This system is composed of sorting channels with actuation electrodes, as well as three trapping chambers each equipped with a wireless, bipolar electrode array. Yeast cells trapped at the center of single bipolar electrodes using negative DEP could be moved along the edges of the electrodes in a noninvasive manner. Similarly, Zaman et al. developed a planar array of sequentially excitable electrodes to induce a moving dielectrophoretic force that allows the bidirectional movement of single particles on a straight line, countering any lateral drift.^[^ ^43^ ^]^ While the suggested electrode geometry was initially optimized for polystyrene beads, the transport of a small cluster of yeast cells (approximately ≈5 μm in radius) was demonstrated.

#### 2.1.2 Magnetophoresis

Magnetophoresis describes the motion of magnetic or magnetized objects in magnetic fields and is used to selectively sort or isolate particles and cells.^[^ ^44^ ^]^ Since cells are not inherently magnetic (with the exception of magnetotactic bacteria), they are most commonly magnetized via magnetic nanoparticle labeling. More modern approaches use magnetogenetics, which focuses on in vivo production and accumulation of magnetic material by cells themselves.^[^ ^45^ ^]^ Alternatively, cells can be suspended in ferrofluids for their indirect manipulation without the need of magnetic labeling, but this method currently has limited applications in integrated microfluidics as biocompatibility of ferrofluids remains a key challenge.^[^ ^46^ ^]^

Similar to dielectrophoretic migration, magnetophoretic migration relies on nonuniform magnetic fields and magnetic susceptibility.^[^ ^47^, ^48^ ^]^ The direction of cell migration in a magnetic field depends on the magnetic susceptibilities of the label particle and the medium. Particles with higher magnetic susceptibility than the medium move toward areas of highest magnetic field strength (positive magnetophoresis).^[^ ^49^ ^]^ Conversely, particles with lower magnetic susceptibility than the medium migrate toward areas of lowest magnetic field strength (negative magnetophoresis).^[^ ^50^, ^51^ ^]^ The magnetic labels used in microfluidics commonly have a larger magnetic susceptibility than the aqueous fluids they are suspended in, resulting in positive magnetophoresis.

To create a magnetic force field across the microchannels, permanent magnets or electromagnetic coils (i.e., electromagnets) are commonly employed. As the strength of a magnetic field rapidly decays with an increasing distance from a magnet, magnets are placed as close as possible to the region of interest. However, this requirement might restrict the integration of multiple workflows on a single device due to geometrical constraints or potential Joule heating effects (e.g., in the use of electromagnets, see Section 2.1.1). To counteract such limitations, recent (integrated) magnetophoretic systems make use of soft magnetic materials to establish localized high magnetic field gradients (and therefore large magnetic forces) in the devices. Soft magnetic materials are materials that can be easily magnetized with relatively weak external magnetic fields and are highly effective in concentrating/altering the applied external magnetic field in their vicinity.^[^ ^49^, ^52^, ^53^ ^]^ The miniaturization and patterning of soft magnetic materials such as NiFe alloys (e.g., permalloy) can be achieved via standard microfabrication techniques, as well as in a simple, low‐cost manner by filling microstructured microcavities with magnetic powders or pastes and capping them with polydimethylsiloxane (PDMS).^[^ ^54^, ^55^, ^56^ ^]^ A recent application that takes advantage of such soft magnetic materials and high gradient magnetic fields is demonstrated by Dumas et al. who microfabricated a NiFe‐based magnetic microtweezer to trap and extract magnetic particles from a continuous stream of droplets (see Section 2.2.4 for droplet microfluidics).^[^ ^57^ ^]^ The platform exhibited exceptional performance in particle extraction, achieving extraction of substantial quantities (10–20 ng) of magnetic particles from droplets of 500 pL. The evaluation of the system's applicability for single‐cell analysis involved mRNA extraction of single cells encapsulated in droplets, resulting in a 43% specific recovery.

**Figure 2:**
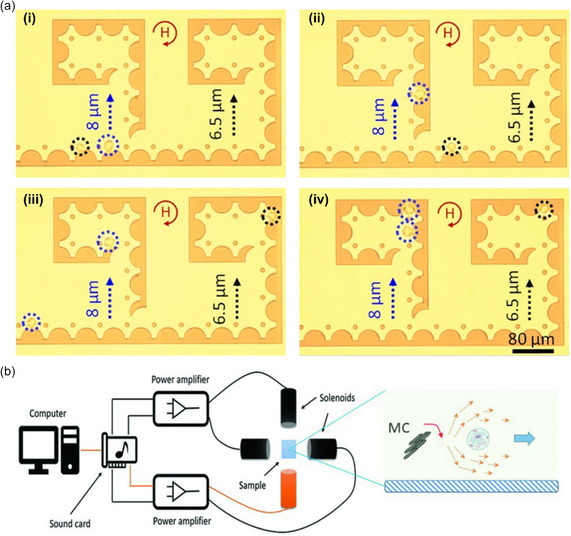
a) Label‐free transportation and storage of single cells in a magnetophoretic circuit. Under rotating magnetic fields (depicted in red), cells suspended in a ferrofluid can be transported along the periphery of magnetic circuit elements and guided to desired cell‐storage locations. Here, the manipulation of unlabeled single human monocytic cells Tohoku Hospital Pediatrics‐1 (THP‐1) of varying sizes (8 μm circled in blue and 6.5 μm circled in black) is shown via bright‐field microscopy images. Adapted under the terms of CC BY 4.0 license.^[^ ^61^ ^]^ Copyright 2021, The Authors. Published by Springer Nature. b) Single‐cell manipulation employing ferromagnetic nanorod clusters actuated by a nonuniform alternating magnetic field. Adapted with permission.^[^ ^62^ ^]^ Copyright 2018, Wiley.

As label‐free systems allow cells to remain in their natural state and are better compatible with downstream separation or analysis methods, negative magnetophoresis has also become a topic of interest for use in integrated microfluidic systems.^[^ ^58^, ^59^ ^]^ One example demonstrated by Liu et al. separated circulating tumor cells (CTCs), the cells of interest, from white blood cells (WBCs) via negative magnetophoresis independent of their physical size and surface antigens.^[^ ^60^ ^]^ In this system, the cell mixture was suspended in a water‐based ferrofluid whose magnetization could be tuned by changing the volume fraction of magnetic materials in the ferrofluid. Unlabeled CTCs were repulsed from the magnet, while nontarget WBCs were magnetically labeled and were attracted by it. In this means, WBCs were negatively selected, allowing for a more efficient separation. A similar yet more advanced application employing negative magnetophoresis was reported by Goudu et al. who could achieve precise, label‐free and high‐throughput sorting, directing, and storing of single cells by suspending them in a biocompatible ferrofluid (biocompatibility investigated up to 4 h) and manipulating them via a magnetophoretic circuit (**Figure** **2**a).^[^ ^61^ ^]^ In another label‐free yet not negative magnetophoresis‐based approach, Zhu et al. developed a method that makes use of ferromagnetic nanorod clusters actuated by a nonuniform alternating magnetic field.^[^ ^62^ ^]^ In contrast to traditional magnetophoresis that employs a permanent magnetic field to manipulate a magnetic/magnetized object, here, the motion of the ferromagnetic nanorods was guided by a nonuniform alternating magnetic field and tuned by additional parameters such as the shape of the nanorods and the frequency of the applied magnetic field (Figure 2b). This flexibility allowed for the pushing, carrying, and dragging of single cells via the nanorod clusters, as well as their release on‐demand. This system could achieve similar translational particle speeds as the ones observed in traditional setups requiring much less power.

#### 2.1.3 Acoustophoresis

**Figure 3:**
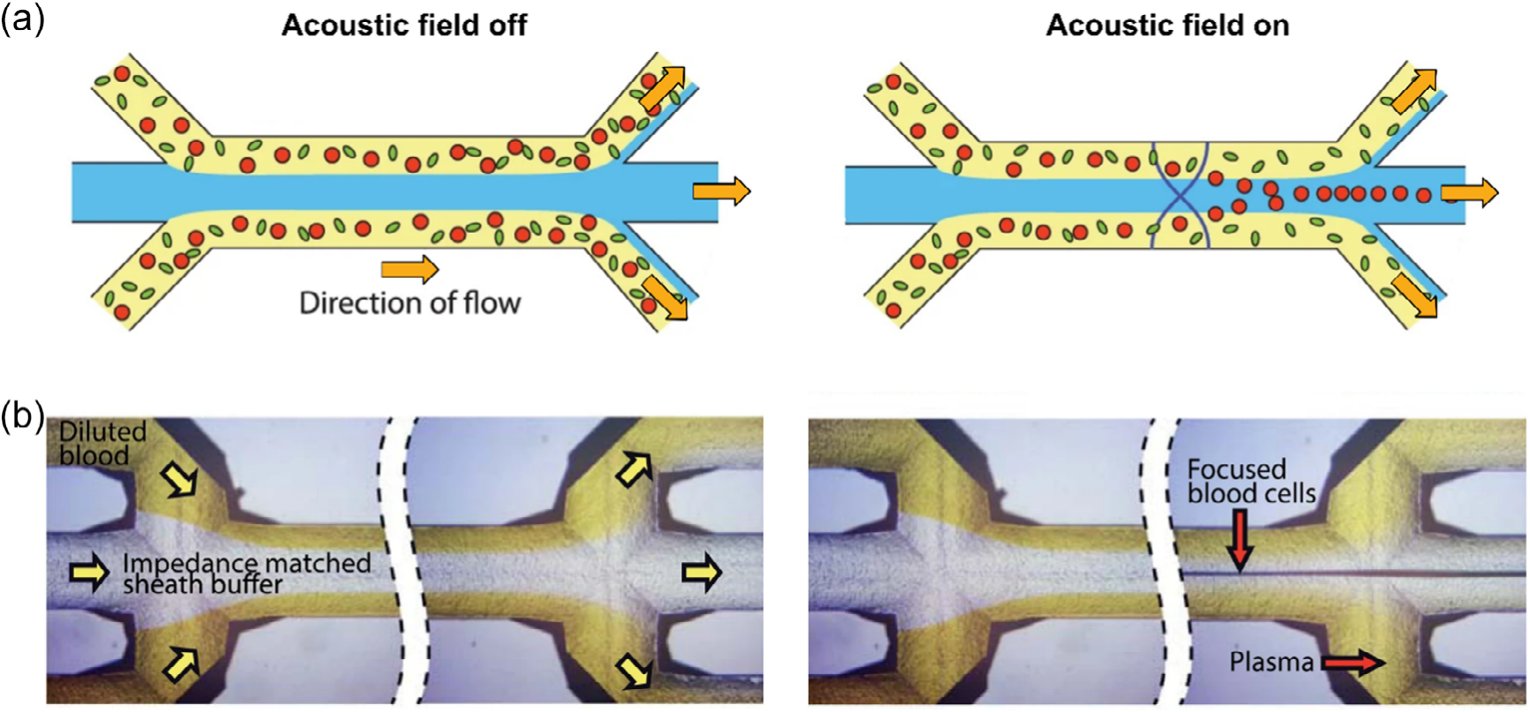
Principle of acoustophoresis. a) When the acoustic field is on, a standing wave (black vertical lines) is generated in the microfluidic channel. While larger cells (here, blood cells, illustrated in red) are pushed by the applied acoustic radiation forces toward pressure nodes, smaller ones (here, bacterial cells, green) are not deflected from the stream by the acoustic field due to their size. b) Microscope images of the microfluidic device whose acoustophoretic separation principle is depicted in (a). By shortening the separation time of blood plasma (yellow) from blood cells, the designed microfluidic device allowed for the quick identification of pathogens in blood. Adapted under the terms of CC BY 4.0 license.^[^ ^247^ ^]^ Copyright 2018, The Authors. Published by Springer Nature.

Similar to electric and magnetic fields, acoustic force fields can also induce a change in the direction of objects. The use of acoustic waves for the manipulation and separation of particles and cells suspended in liquids is called acoustophoresis. Acoustophoresis is a non‐contact, label‐free method that provides good spatial control and high sorting resolution.^[^ ^63^ ^]^ When sound waves resonating inside microfluidic devices are reflected at the channel walls, they establish standing waves, which generate strong pressure gradients steering cells/particles toward specific positions in the channel.^[^ ^64^ ^]^ This separation relies on intrinsic cell/particle properties, such as density, compressibility, and size, as well as the material properties of the acoustophoretic chip and the geometry of the microchannel.^[^ ^65^, ^66^ ^]^ As most cells and microparticles are denser or less compressible than their surrounding medium, they have a positive acoustic contrast factor (*ϕ* > 0 see Table S1, Supporting Information) and are therefore steered toward pressure nodes, i.e., regions of lowest pressure amplitude.^[^ ^67^ ^]^ Since (primary) acoustic radiation force is proportional to the volume of an object, larger cells/particles experience larger acoustic radiation forces, which enables size‐selective separation (**Figure** **3**a). Once cells/particles are positioned with acoustophoresis in a microfluidic channel, they are held in the respective fluid streams by the laminar flow, which facilitates sorting into different outlets (Figure 3b).

Acoustophoresis commonly requires the presence of a bulk piezoelectric transducer that creates acoustic waves through the microchannel, or the integration of interdigitated piezotransducers that create surface acoustic waves propagating along solid–fluid or solid–air interfaces.^[^ ^68^ ^]^ The use of bulk piezoelectric transducers limits the number of workflows that can be integrated into a single microsystem due to geometrical constraints, restricting their use in integrated microfluidics. To overcome such limitations regarding materials and parts miniaturization, Antfolk et al. demonstrated a plug‐and‐play system composed of a glass acoustophoretic sorting chip and a DEP‐based isolation chip bonded to each other for the efficient trapping and enumeration of prostate cancer cells.^[^ ^69^ ^]^ However, integrated systems today are typically designed to execute comprehensive operations within a single chip, typically fabricated using polymers. This poses a significant challenge for the use of acoustophoresis in integrated platforms, as acoustophoretic separation requires microchannels made of materials with high specific acoustic impedances relative to the fluid (e.g., silicon or glass).^[^ ^70^ ^]^ In this front, multiple researchers are reporting numerical and experimental work to widen the applications of acoustophoretic principles in polymer‐based platforms.^[^ ^66^, ^70^, ^71^, ^72^ ^]^ In addition, advanced acoustophoretic applications that can enhance particle separation and manipulation are explored in an increasing manner,^[^ ^73^, ^74^ ^]^ some examples being size‐independent cell separation via isoacoustic focusing,^[^ ^75^ ^]^ generation of 2D acoustic standing waves in microchannels that help focus cells of nonuniform shape (e.g., ellipsoidal versus spherical),^[^ ^76^ ^]^ and acoustic cell washing, which can help integrate cell washing steps into integrated systems.^[^ ^77^, ^78^ ^]^ Further technologies of interest include acousto‐dielectric tweezers, which can achieve high‐throughput and size‐independent manipulation and biophysical analyses of single cells, or acoustic streaming, which can perform a series of precise single‐cell manipulations, including rotation, selective trapping, controllable release, and particle pairing.^[^ ^79^, ^80^, ^81^, ^82^ ^]^

Secondary acoustic radiation forces, which are responsible for particle–particle interactions in an acoustic field, are also explored to manipulate single‐cell motion in microfluidic channels.^[^ ^83^ ^]^ The magnitude and direction of secondary acoustic radiation forces have been found to strongly depend on the orientation of the particle pair relative to the wave propagation direction.^[^ ^84^ ^]^ Saeidi et al. investigated these forces acting between cells and immobilized silica particles in an ultrasonic standing wave generated in a microfluidic channel. The authors showed that in an acoustic field, cells in close proximity to immobilized silica particles can be deflected in the transverse direction relative to wave propagation. They also found that the secondary acoustic radiation force can overcome the primary acoustic radiation force, resulting in red blood cells (RBCs) being trapped by the silica particles at positions in between a pressure node and a pressure antinode. Although single‐cell trapping was not observed when this method was applied to larger human adenocarcinoma cells Michigan Cancer Foundation‐7 (MCF‐7), ongoing research into acoustic principles is expected to yield innovative applications for integrated workflows at the single‐cell level.

#### 2.1.4 Optical Tweezers

Optical tweezers use tightly focused laser beams to create optical traps that allow the manipulation of neutral, transparent microscopic objects, including living cells. In single‐cell research, optical tweezers can be utilized for a variety of processes, including cell positioning, transportation, assembly, and sorting.^[^ ^85^, ^86^ ^]^ The principle behind optical tweezers lies in the fact that optically transparent objects, such as cells, can be manipulated (i.e., moved, trapped, or stretched) using light diffraction and reflection, due to a change in momentum. As momentum is fundamentally conserved, some momentum is transferred from the light to the diffracting/reflecting cell/particle, exerting a force onto it.^[^ ^87^, ^88^ ^]^ The forces involved in optical trapping stem from photons scattering when hitting a cell/particle and the gradient force arising from the intensity distribution of the laser light used for manipulation.^[^ ^86^ ^]^ Scattering and gradient forces depend on the cell/particle size relative to the wavelength of the laser beam and can be calculated assuming the created optical trap is a harmonic oscillator (see Table S1, Supporting Information). Optical trapping usually takes place in two dimensions, as optical force gradients are not strong enough to allow manipulation and trapping in 3D.^[^ ^85^ ^]^ To overcome this limitation, optical setups that enhance the stiffness of optical traps have been developed.^[^ ^89^ ^]^

Despite the flexible application opportunities optical tweezers offer, in practice, the method still commonly requires manual cell manipulation by skilled personnel and expensive optical setups, which limits throughput, broad adoption, and scalability.^[^ ^90^, ^91^ ^]^ One way to overcome such limitations might be the use of optofluidic devices which use miniaturized optical components like light sources, lenses, and waveguides that are integrated into the microfluidic devices.^[^ ^92^, ^93^ ^]^ In 2013, Liberale et al. miniaturized fiber‐based optical tweezers to achieve stable 3D single‐cell trapping in a microfluidic system.^[^ ^94^ ^]^ To create the optical trap, microprisms were fabricated on the fiber facets using two‐photon lithography, and the capabilities of the device were demonstrated using fluorescence and Raman spectroscopy measurements performed on trapped RBCs and colon cancer cells. Advances in the miniaturization of optical elements and their application to optical tweezers has been recently reviewed.^[^ ^95^ ^]^ Furthermore, there is an accelerating interest in utilizing multiple physical principles simultaneously to realize optically induced trapping, one prominent example being optoelectronic tweezers (OET).^[^ ^90^ ^]^ By combining photonics and electronics, OET projects programmable light patterns onto a photoconductive substrate creating nonuniform electric fields for sample manipulation. Compared to optical tweezers, OET allows for massive parallelization (e.g., 10 000 traps) and the capability of manipulating objects ranging from tens of nanometers to several hundred micrometers in size.^[^ ^90^ ^]^ Additionally, OET operates using simpler equipment (i.e., LED light sources and digital micromirror devices) and lower light power density, making it more accessible and suitable for handling heat‐sensitive biological samples.^[^ ^90^ ^]^

### 2.2 Passive Cell Sorting and Isolation Methods—Introduction of Methods and Novel Applications

#### 2.2.1 Inertial and Hydrodynamic Focusing

Inertial and hydrodynamic forces acting on neutrally buoyant objects, such as cells suspended in a laminar flow, can be exploited to manipulate their position. Inertial focusing makes use of cross‐stream particle motion to concentrate particles into one or multiple streams.^[^ ^96^ ^]^ Suspended cells/particles move in the direction of flow as a result of the viscous drag forces arising due to the velocity difference between them and the surrounding fluid. In the direction orthogonal to flow, the position of cells/particles is primarily determined by the equilibrium between wall‐ and shear gradient‐lift forces, which push them toward the channel centerline and the channel walls, respectively.^[^ ^97^, ^98^ ^]^ In curved channels as well as in straight channels with disturbance structures (e.g., structures that disrupt the homogenous channel cross‐section geometry), the lateral position of cells/particles is additionally affected by secondary‐flow drag forces.^[^ ^98^, ^99^ ^]^ Secondary flow, also called rotational flow or Dean flow, occurs due to the velocity mismatch between fluids at different cross‐sectional regions as a result of the inertia of the fluid itself.^[^ ^98^, ^100^, ^101^, ^102^ ^]^ As Dean flow is oriented perpendicular to the main flow, it also exerts a drag force on the submerged cells/particles. By manipulating these forces, cells and particles of varying properties can be steered into different lateral equilibrium positions in laminar streams, which facilitates inertial focusing and therefore sorting.

Hydrodynamic focusing in microfluidics leverages the differences in flow rates and velocities of fluid streams within a microchannel to achieve a focusing effect. In this process, a main fluid, which contains cells or particles, is directed along the center of the channel and laterally compressed toward this central plane by two sheath fluid streams flowing through adjacent side channels.^[^ ^96^ ^]^ The sheath fluids move at higher velocities compared to the main fluid, resulting in a velocity profile that guides and confines the main fluid into a narrow stream at the center. While this technique effectively focuses the cells or particles in the lateral dimension (width) of the channel, it may not adequately address their distribution in the vertical dimension (depth). This vertical dispersion can adversely impact system performance in applications such as imaging or cell counting, where precise positioning is crucial.^[^ ^96^, ^103^ ^]^ To mitigate this limitation, 3D hydrodynamic focusing has been developed and is becoming increasingly prominent. This advanced method enables control over the positioning of cells or particles in both the horizontal and vertical dimensions of the channel, commonly by manipulating the flow rates and velocities of multiple sheath fluid streams around the main fluid.^[^ ^103^, ^104^ ^]^

In the context of integrated microfluidics, inertial and hydrodynamic focusing allows the development of simple, compact, and low‐cost platforms. Cell manipulation takes place in a label‐free manner, as steering forces only rely on the geometry of the microfluidic channel, the Reynolds number, and the geometry, size, and density of the cell/particle.^[^ ^102^, ^105^ ^]^ However, cell properties are not always known, for example in the case of clinical samples. This can negatively affect the separation efficiency of the platform, or requires extensive optimization and preliminary sample analysis. Furthermore, devices might experience clogging especially when a high density of cells are present (e.g., whole blood processing).^[^ ^106^ ^]^

#### 2.2.2 Microarchitecture‐Based Separation ‐ Micropillars and Deterministic Lateral Displacement

**Figure 4:**
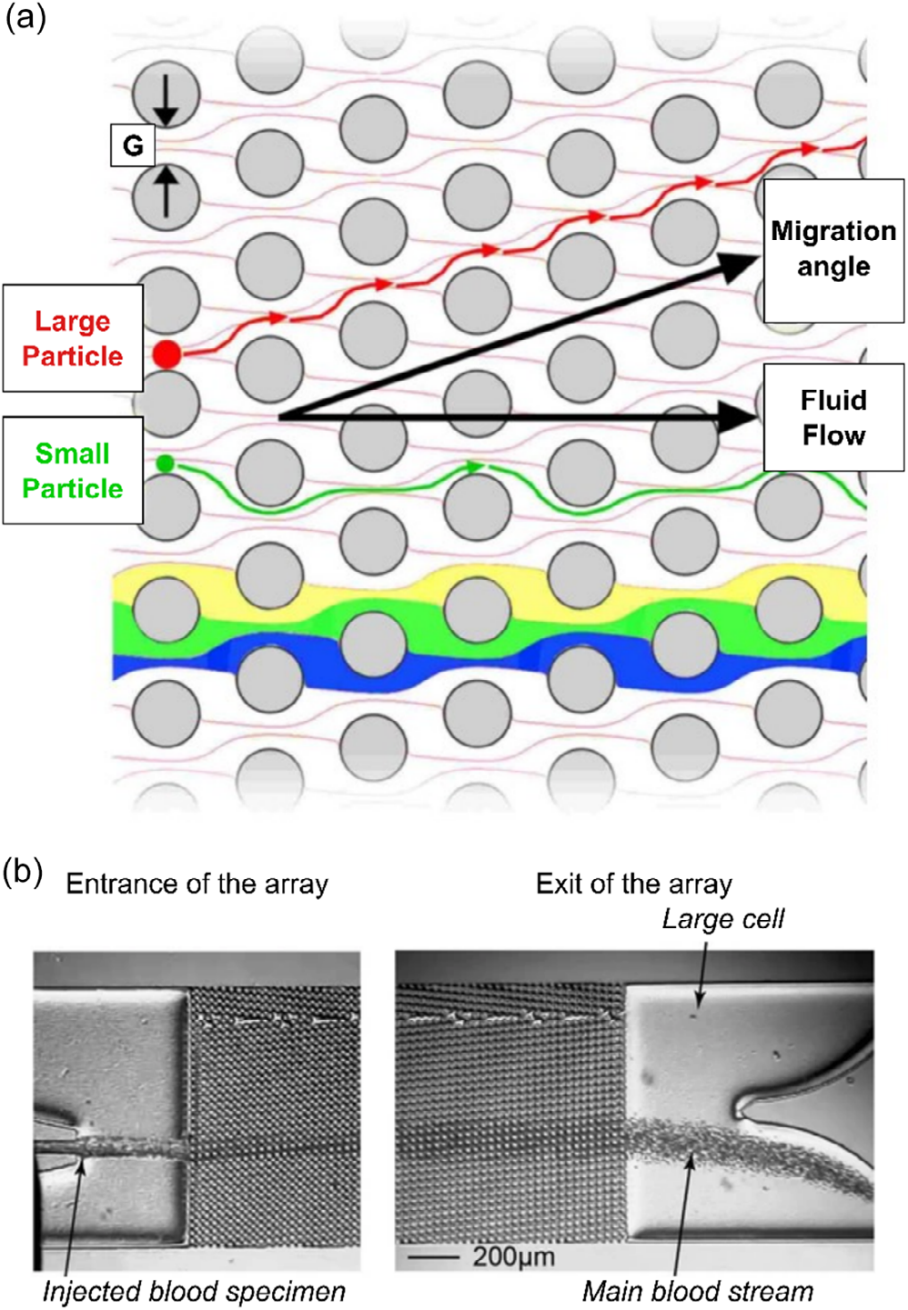
Working principle of DLD. a) DLD relies on the asymmetric bifurcation of laminar flow around obstacles. In this illustration, periodic flow patterns (i.e., streamlines) observed in a DLD array are highlighted in blue, green, and yellow. Cells/particles entering a DLD array are separated according to their size by slightly offsetting repeating rows of obstacles/pillars in a microfluidic channel. Adapted with permission.^[^ ^248^ ^]^ Copyright 2009, AIP Publishing. b) A microscope image of a microfluidic DLD array used to separate blood cells based on their size. When diluted mouse blood was injected into the array, large cells could be seen exiting the device through the upper outlet, while the main blood stream exited through the bottom outlet. Adapted with permission.^[^ ^249^ ^]^ Copyright 2015, Springer Nature.

Micropillars are custom‐microstructured arrays of varying sizes and geometries that find multiple uses in microfluidic devices, such as sorting of cells and particles from heterogeneous populations (discussed in this section), or capturing and isolation of single cells and cellular components when utilized as traps (discussed in Section 2.2.3). The most prominent use of micropillars in cell sorting based on size and deformability is deterministic lateral displacement (DLD). Unlike traditional micropillar arrays, which commonly act as a filter and only allow small objects to pass through while blocking large ones, DLD micropillar arrays allow objects of various sizes to pass.^[^ ^107^ ^]^ In DLD‐based cell sorting, cells/particles with radii smaller than a critical radius pass through the intercolumn gaps and move along the main flow direction. Cells/particles with radii larger than the critical radius migrate laterally to adjacent streamlines determined by the shape and the geometry of the micropillar array (**Figure** **4**a).^[^ ^11^, ^108^, ^109^ ^]^ In this manner, continuous sorting of cell populations, organelles, or particles is possible (Figure 4b). Significant advantages of DLD‐based sorting include a high resolution (a separation limit down to 20 nm) and a high dynamic size‐separation range, which spans from millimeter‐ to nanometer‐sized objects.^[^ ^110^ ^]^

#### 2.2.3 Microarchitecture‐Based Separation—Microwells, Microchambers, and Microtraps

Among the passive cell separation methods, (functionalized) microwells, microchambers, and microtraps are particularly popular for single‐cell isolation/immobilization as well as for (long‐term) analysis of cellular activity and cell–cell interactions on‐chip. Depending on the application, the geometry of these structures needs to be optimized to ensure reliable filling, prevent bubble formation, and avoid the entrapment of multiple cells in a single unit when single‐cell analysis is intended.^[^ ^111^, ^112^ ^]^ In addition, the properties of the cell types (i.e., adherent or suspension), as well as the mechanisms of nutrient supply (i.e., diffusive and convective), should be considered when selecting a suitable architecture.^[^ ^113^ ^]^ Micropillars can also be utilized as microtraps, their biggest advantage being the high surface‐area‐to‐volume ratio, which can lead to an improved adhesion of cells when combined with affinity‐based approaches.^[^ ^114^ ^]^ While microwells, microchambers, and microtraps can be used as standalone structures for cell separation, to enhance the performance of integrated microfluidics, they are often combined with other cell separation methods, such as dielectrophoretic trapping or magnetophoresis. Furthermore, the use of such microunits in combination with miniaturized biological assays allows significant advances in single‐cell analysis capabilities, as discussed in multiple examples in Section 4.

A novel application demonstrating the potential and versatility of microwells for cellular analyses was recently reported.^[^ ^115^, ^116^ ^]^ In these works, the authors used a non‐sealed set of stackable microwell arrays, called “Stacks”, for the controlled design of the complex tumor microenvironment. The microwell design allowed for open and suspended microfluidics, leveraging surface tension to prevent liquid loss. Cells added to individual microwells in a specified volume of culture media could be stacked on top of each other without leakage, creating a unique vertical communication pathway across aligned wells. Furthermore, it was possible to mimic the extracellular matrix (ECM) properties by adding matrices such as collagen or fibronectin into the wells before cell seeding. The incorporation of open microfluidic channels into each stack‐enabled fluid handling. While this platform has not been demonstrated for the isolation of single cells, it can inspire the design of future single‐cell cocultures in integrated microfluidic platforms.

#### 2.2.4 Droplet‐Based Microfluidics

Microscale droplet generation and manipulation has been one of the most influential tools for microscale analyses, leading to the development of multiple powerful technologies such as digital droplet polymerase chain reaction, single‐cell RNA sequencing, and digital microfluidics (DMF).^[^ ^117^, ^118^, ^119^ ^]^ In microfluidic channels, droplet formation under continuous flow typically involves the manipulation of immiscible phases, such as water and oil, at microfluidic junctions and flow‐focusing geometries.^[^ ^120^, ^121^, ^122^ ^]^ For single‐cell workflows, this method is used to encapsulate individual cells in monodisperse droplets ranging from femtoliters to nanoliters, often termed “microdroplets”. Once single cells are encapsulated in microdroplets, they can be sorted into various outlets for downstream cell culture and/or characterization.^[^ ^123^ ^]^ In contrast to flow‐based systems, technologies such as DMF typically operate under no flow conditions. In DMF, droplets are precisely manipulated (e.g., split, moved, merged) on a hydrophobic surface by means of different actuation principles. The most common actuation principle is electrowetting on dielectric, which causes a reversible wettability shift induced by an electric charge on a dielectric surface.^[^ ^124^ ^]^ Tong et al. have recently reviewed system‐level and chip‐scale integration of DMF devices, as well as hybrid actuation strategies, including digital‐to‐channel and channel‐to‐digital interfaces.^[^ ^124^ ^]^

**Figure 5:**
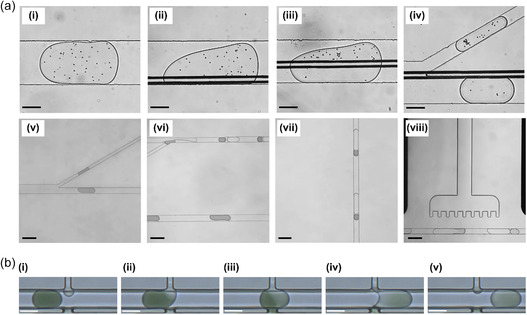
Methods developed to exchange droplet contents. a) Operation steps of the in‐droplet washing system demonstrated using *Chlamydomonas reinhardtii* cells and color dye. i–iv) Cells encapsulated in the droplet are strategically relocated before droplet splitting using DEP. Scale bar: 100 μm. v–vii) The top daughter droplet cleaves the continuous stream of fresh solution, resulting in one‐to‐one droplet pairs. viii) Paired droplets are merged under an electric field. Scale bar (v–viii): 200 μm. Adapted with permission.^[^ ^133^ ^]^ Copyright 2021, American Chemical Society. b) Simultaneous liquid addition and removal into/from droplets without droplet splitting–merging steps using Pico‐washer. The high‐pressure washing and low‐pressure waste streams can be seen at the top and bottom of the emulsion channel, respectively. Adapted under the terms of CC BY 4.0 license.^[^ ^134^ ^]^ Copyright 2022, The Authors. Published by Springer Nature.

Droplet‐based microfluidic technologies and their application to single‐cell workflows offer significant advantages such as high throughput, parallelization possibilities, controlled confinement of single cells, and significant reagent cost reduction.^[^ ^125^, ^126^, ^127^ ^]^ Furthermore, droplets provide an ideal microenvironment for conducting a variety of biological assays, offering precise spatial and temporal resolution. For example, multiple recent publications have showcased the substantial analytical potential of DMF for single‐cell genome and transcriptome analyses.^[^ ^128^, ^129^, ^130^ ^]^ However, numerous challenges remain that limit the wide application of droplet microfluidics, including the nondeterministic distribution of cells into microdroplets that results in a high number of empty droplets.^[^ ^131^, ^132^ ^]^ To counteract this limitation and minimize inefficient operation, Yu et al. recently reported a smart droplet‐based microfluidic system with integrated image recognition for selective droplet manipulation and real‐time sorting.^[^ ^131^ ^]^ Another considerable drawback is small droplet volumes, as cell viability and long‐term cell culture possibilities are restricted due to limited nutrient supply and waste removal. Various approaches have been developed to overcome this challenge. Huang et al. introduced an in‐droplet cell washing and solution exchange system, using DEP to strategically relocate cells within droplets before splitting and merging them with a new medium, replacing up to 88% of the original medium with minimal cell loss (**Figure** **5**a).^[^ ^133^ ^]^ Similarly, Siedlik et al. created a “Pico‐washer” that can exchange the contents of a droplet under flow via the simultaneous addition and removal of fluids into/from it. This system exploited the differential pressure between droplet washing (high‐pressure) and waste (low‐pressure) streams, as well as the application of an alternating electric field at the washing region to destabilize the water–oil interfaces at the point of contact, thereby facilitating the transfer of fluids (Figure 5b).^[^ ^134^ ^]^ While this system was not demonstrated for use with cells, the authors used beads to investigate the microparticle retention in droplets during washing. A bead retention of 70% could be achieved with a dilution factor of 2.5 at throughputs between 0.5 and 1.0 kHz. A different approach was demonstrated by Isozaki et al. who cultivated single cells in large droplets (≈100 pL) in contrast to traditional small droplets (≈10 pL).^[^ ^135^ ^]^ As larger droplets are easier to break, the authors developed a dielectrophoretic array able to handle them efficiently without breakage, using sequential electrode activation for gentle manipulation.^[^ ^135^ ^]^ This method improved cell viability in various cell lines, including human T lymphocytes. However, large droplets are generally less suitable for high‐throughput sorting and most microfluidic systems are limited in the range of droplet sizes they can generate and handle, which might limit the wide adoption of large droplet‐based approaches.^[^ ^122^, ^135^ ^]^

While droplet characteristics can be precisely tuned to accommodate various cell types, establishing adherent single‐cell cultures in droplets remains a challenging task.^[^ ^136^ ^]^ Adherent cells need to attach onto supporting substrates via cell–ECM interactions to prevent anoikis (i.e., cell death induced by inadequate or inappropriate cell–matrix interactions).^[^ ^137^, ^138^ ^]^ As hydrogels are often used as ECM‐mimicking environments, single adherent cells can be encapsulated into hydrogel droplets. However, this method has technical challenges such as throughput, uneven hydrogel formation, or possible cytotoxicity.^[^ ^121^, ^139^ ^]^ To overcome this limitation, Wang et al. initially anchored adherent single cells onto microfabricated gelatin particles, and then encapsulated this cell–hydrogel microparticle duo in droplets.^[^ ^140^ ^]^ In this setup, droplets did not have to go through a polymerization step after generation; therefore cell cytotoxicity was minimized. Furthermore, it was possible to tune the mechanical characteristics (i.e., stiffness) of gelatin particles, providing a mechanically tunable microenvironment for single cells.

One further challenge with droplet microfluidics is associated with the lack of precise control of droplet locations and the selective, dynamic manipulation of droplets under flow conditions. To address this challenge, Sun et al. used microwells, termed “Surface Energy Wells”, embedded in microchannels for the dynamic manipulation of single droplets.^[^ ^141^ ^]^ In this platform, generated droplets were squashed into a flattened shape by the reduction of channel height, which increased the droplet surface energy. When droplets encountered a microwell (i.e., a channel section with a larger height), they could be temporarily confined at the embedded microwells due to a release in surface energy. This method has not only allowed for single‐droplet trapping, but also for the controlled replacement of droplets in as little as 3 s by changing the flow rate of the carrier oil. Another example was demonstrated by Liu et al. who utilized a coating of fluorinated magnetic nanoparticles over the surface of the cell‐encapsulating droplets to manipulate their position under applied magnetic fields.^[^ ^142^ ^]^ The innovative aspect of this approach was its ability to preserve high biocompatibility within the droplets by preventing direct contact between the magnetic particles and the encapsulated cells, while simultaneously ensuring that the droplets exhibit a strong response to the magnetic field. In a different approach, Park et al. demonstrated a “DMF‐like” manipulation technique that can be used under continuous flow by employing individually addressable ion permselective membranes, i.e., membranes that selectively allow certain types of ions to pass while blocking others.^[^ ^143^ ^]^ The application of an electric field across the ion permselective membranes induced ion‐enriched or ion‐depleted layers at the membrane–fluid interfaces. Biomolecules carrying a charge in a microfluidic channel accumulated at the interface of the ion‐depleted zone, forming concentrated areas of biomolecules termed “plugs”. By varying the electric field across the membranes, the movement of these plugs could be controlled leading to their translation, merging, and splitting, similar to DMF operations.

## 3 Methods for Single‐Cell Analysis in Integrated Microfluidics

Cellular analysis strategies can be broadly grouped into three categories: 1) biological analysis of whole cells without integrity disruption; 2) biochemical analysis following cell lysis; and 3) total single‐cell analysis, which combines biological analysis followed by biochemical analysis.^[^ ^144^ ^]^ Biological analysis is commonly noninvasive and includes the study of cell morphology (e.g., size, shape), cell movement, and cell vitality. Biochemical analysis commonly requires the disruption of cellular integrity to study intracellular components (e.g., genomic material, proteins, and organelles). As we discuss later, biological and biochemical analyses performed on‐chip are mainly achieved optically using microscopes or spectrometers, electrically via integrated microsensors or external sensors, or mechanically using microstructures or force fields. For all methods, it is generally desirable that analysis is performed at high speed and high resolution, with high specificity and sensitivity, and low reagent and sample consumption.

### 3.1 Optical Analysis of Cells in Integrated Microfluidics

Optical cell analysis plays a pivotal role in cellular studies, offering a diverse set of techniques to explore cellular structures, behavior, and function. Phenomena like absorbance, fluorescence, luminescence (e.g., chemiluminescence), and scattering (e.g., Raman scattering) can reveal information both in cellular and molecular levels, enabling various strategies for biological and biochemical analyses of single cells.^[^ ^145^ ^]^ Due to the indispensable nature of optical analysis, microfluidic systems are most often fabricated using transparent materials, commonly glass or PDMS, and are compact enough to be compatible with the use of microscopy. Bright‐field microscopy is a foundational optical technique that is commonly utilized to obtain an initial set of information (e.g., cellular morphology) from a given sample. However, as cells are composed mainly of water, they are virtually transparent, which complicates their imaging under basic bright‐field illumination. These limitations can be countered by cell staining to enhance visibility and contrast, but this procedure is often invasive and can disturb or alter the physiological state of cells. Alternatively, biological investigations related to cell vitality, integrity, morphology, or growth can be carried out without cell staining via high‐contrast bright‐field microscopy techniques such as phase contrast imaging. However, for many analysis goals, the overall contrast and the specificity offered by bright‐field microscopy are not adequate. Therefore, fluorescence microscopy, while commonly requiring cell fixation, permeabilization, and/or labeling/tagging, remains a crucial optical cell analysis method for a wide range of analyses, such as identification of cells and cellular components and tracking cellular expression profiles or protein localizations, demonstrated in multiple examples in Section 4. In contrast to single‐workflow microfluidic systems, which commonly use fluorescence imaging for cell sorting (e.g., fluorescence‐activated cell sorting), integrated microsystems tend to use fluorescence imaging as a final analysis method following cell sorting and isolation to maximize the amount and intensity of fluorescent dyes available for analysis and minimize the potential alterations in cell biology due to fluorescence tagging. Both bright‐field and fluorescence microscopy are often combined with cell focusing and/or trapping so that individual cells can be better imaged. Furthermore, optical cell analysis is an integral part of mechanical characterization of single cells, as mechanical property evaluation often relies on changes in cell shape recorded using microscopy (see Section 3.3).

**Figure 6:**
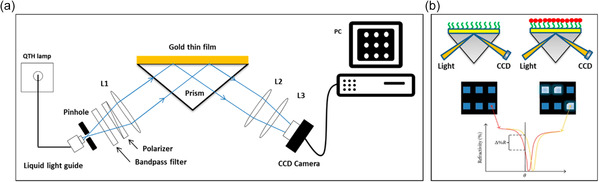
General principle of SPR imaging. a) The light beam is focused on a prism coupler and is reflected from the SPR sensor surface (here, gold). Reflected light is captured by a monochromatic CCD camera. b) Analyte–ligand interactions at the sensor surface cause a change in its reflectivity and a shift in the SPR curve. Adapted under the terms of CC BY 4.0 license.^[^ ^147^ ^]^ Copyright 2015, The Authors. Published by MDPI.

In addition to bright‐field and fluorescence microscopy, spectroscopy‐based microscopy methods such as Fourier transform infrared (FTIR) spectroscopy, Raman spectroscopy, and surface plasmon resonance (SPR) can provide crucial information on a molecular level for the label‐free biochemical analyses of single cells.^[^ ^145^ ^]^ The power of these methods stems from the creation of images that can convey spectroscopic data in a spatially resolved manner. However, when integrating these analysis methods into microsystems, factors such as microfabrication complexity, microsystem material, equipment compatibility with established protocols, and possible interference due to biological sample properties should be considered. For example, the application of SPR‐based imaging on‐chip commonly requires the manufacturing of specialized surfaces as sensing layers (e.g., ligand‐bound metal layers) that are interfaced with high‐refractive‐index prisms, which reflect the incident light onto a monochromatic charge‐coupled device (CCD) camera (**Figure** **6**).^[^ ^146^, ^147^ ^]^ This setup complicates the integration of SPR sensors into integrated microfluidics to perform SPR‐based imaging. One way to overcome this limitation was recently demonstrated by Debnath et al. where a plug‐and‐play system configuration was used to connect a blood‐plasma‐separation chip to a commercial SPR sensor chip via microfluidic tubing.^[^ ^148^ ^]^ This system combination allowed for the processing of whole blood samples in the separation chip and the detection of IgG and IgM antibodies in the SPR sensor chip in less than 15 min. Another system reported by Xiao et al. used 3D‐printing and adhesive tape‐based sealing to realize a microfluidic platform that could be directly placed on top of the sensing layer of a commercial SPR sensor.^[^ ^149^ ^]^ In this work, the authors used a smartphone based optical readout and demonstrated the detection of ß2 microglobulin, a protein used as a biomarker for various disease states, spiked in a running buffer optimized for SPR applications. A similar challenge exists for FTIR‐based microscopy, which finds limited applications due to the short penetration depths (≈ a few micrometers) of infrared (IR) light into the samples, as the aqueous media commonly used for cell‐related applications in microfluidic devices absorb IR strongly.^[^ ^150^ ^]^ In response to this significant challenge, the attenuated total reflection FTIR (ATR–FTIR) spectroscopy method has been introduced. ATR–FTIR involves using an optically dense material, like silicon, as an internal reflection element (IRE or ATR crystal) that creates an evanescent wave on its surface. Employing the surface of the ATR crystal as a substrate for the microfluidic systems makes it possible to directly interrogate samples resting on the IRE.^[^ ^150^, ^151^ ^]^ However, the necessity to build the microfluidic platforms atop of an optically dense IRE/ATR crystal presents challenges in terms of miniaturization, materials that can be used for microfluidic device construction, and optical analysis capabilities. Recently, Jia et al. reported a novel design that embeds a miniaturized multi‐groove silicon ATR crystal into microfluidic devices made out of PDMS. This design alleviated the spatial limitations associated with the use of the ATR crystal as the structural element, thereby offering enhanced design flexibility.^[^ ^152^ ^]^ Such advancements could pave the way for more effective integration of FTIR‐based methods into integrated microfluidics.

### 3.2 Electrical Analysis of Cells in Integrated Microfluidics

Although microscopy has been the major driver of progress in biology for decades, conventional microscopy methods, such as fluorescence imaging, require sample processing that might disturb or alter the physiological state of cells, as discussed earlier. To overcome this limitation, electrical cell analysis methods have been established as a noninvasive and label‐free alternative to optical analysis. To characterize the electrical properties of whole cells, equivalent circuit models have been proposed.^[^ ^153^ ^]^ In these models, electrical characteristics of the cell membrane, cellular contents, and the surrounding medium are taken into consideration.^[^ ^154^ ^]^ For example, a cell membrane can be electrically represented by a combination of a capacitor and a resistor. While intact cell membranes are poor conductors, damaged membranes leak ions and therefore become conductive. Thick membranes and membranes of high morphological complexity have higher capacitance and therefore require longer charging times; whereas thinner membranes are charged faster. Similarly, the cytoplasm enclosed within the cell membrane is considered a conductive element, whose conductivity can be affected by various processes such as apoptosis, progression of cancer, and stem cell differentiation.^[^ ^153^, ^155^ ^]^

When it comes to on‐chip electrical cell analysis, integrated microelectrodes play an important role. Miniaturized electrodes have been incorporated into microfluidic devices for over a decade using a variety of techniques including wire insertion, sputtering, evaporation, and photolithography.^[^ ^153^ ^]^ The challenges associated with integrated electrodes are primarily related to electrode polarizability and material stability in liquid environments. Contact with ionic liquids often causes the formation of dielectric double layers at the metal/liquid interface, giving rise to electrode polarization effects.^[^ ^153^ ^]^ Additionally, redox electrochemical reactions can be induced due to charge transfer across the interface, resulting in faradaic current. As such processes can have a significant effect on the experimental data, it is of outmost importance that suitable electrode materials are selected for the intended applications. For example, for AC and high‐frequency measurements, polarizable electrodes that allow no faradaic current flow across the interface, such as Au and Pt, are preferable. In contrast, the presence of faradaic current is an intrinsic necessity for DC and low‐frequency measurements for current conduction, and therefore non‐ or low‐polarizable electrodes made from materials such as Ag are used.^[^ ^153^ ^]^ Additional electrode properties to be considered include chemical stability in physiological buffers, biocompatibility, and optical transparency, especially if simultaneous optical analysis is desired.

#### 3.2.1 Microelectrical Impedance Spectroscopy in Integrated Microfluidics

In single‐cell analysis, microelectrical impedance spectroscopy (μ‐EIS) is most commonly used to monitor the electric current response of trapped cells when subjected to an AC field applied across the trap volume using integrated microelectrodes. The method relies on the frequency‐dependent dielectric properties of cells which arise from various cell polarization mechanisms. For example, low‐frequency dispersion (also known as α dispersion, 1 Hz–100 kHz) has been found to arise from cell membrane permeability due to ion transport. β dispersion (100 kHz to several MHz) is associated with the interfacial polarization and morphology of cellular membranes. High‐frequency dispersion (γ dispersion (>10 GHz)) is primarily associated with polarization due to the reorientation of water molecules and is therefore normally not investigated when characterizing cells.^[^ ^153^ ^]^ To obtain an impedance spectrum that captures the electrical characteristics of cells across varying frequency ranges, a frequency sweep is carried out. Various models have been developed to analyze such data, which have been primarily used to differentiate between various cell types or to monitor cell heterogeneity after cell isolation.^[^ ^156^ ^]^ For example, time‐lapse cell cycle analysis was conducted on *Schizosaccharomyces pombe* yeast cells in an integrated microfluidic device that combined single‐cell immobilization, μ‐EIS, cell culture, and microscopy. A suction channel was used to pull yeast cells into microtraps located between an excitation and a measuring electrode. Impedance was monitored to determine cell size along the cell cycle and was verified using confocal microscopy.^[^ ^157^ ^]^ In another example, Mansoorifar et al. developed a lab‐on‐chip device that uses an electro‐activated microwell array for capturing single cells, impedance measurements, and cell unloading. An equivalent circuit model was developed to extract the cell membrane capacitance and cytoplasmic conductivity from the impedance spectra.^[^ ^158^ ^]^ In this example, cells were isolated at microelectrodes via dielectrophoretic trapping. Real‐time measurements of dielectric properties of live cancer cells were performed and cellular responses to variations in buffer conductivity and pH were assessed. Although a wealth of information can be extracted from analyzing EIS data, the method has the disadvantage of requiring complex theoretical models for data analysis. Moreover, single‐cell EIS has an inherently low throughput, which stems from the required cell trapping and releasing process, especially compared to impedance cytometry approaches that have been shown to be capable of cell interrogation rates greater than 100 cells s^−1^.^[^ ^159^ ^]^

#### 3.2.2 Dielectrophoretic Cell Analysis in Integrated Microfluidics

In addition to cell sorting and isolation, DEP can also be utilized as a label‐free cell analysis method due to the strong correlation between cell electrophysiology and the dielectrophoretic force.^[^ ^160^ ^]^ However, it is not possible to directly measure the DEP force. Instead, several methods have been established to measure cell (or particle) velocity, as it is directly proportional to the DEP force. One such indirect method used to characterize cells measures the so‐called crossover frequency, i.e., the AC frequency at which the polarizability of the particle is identical to that of the suspending medium, at which there is no induced dipole and the dielectrophoretic force is zero. The crossover frequency has been shown to depend on various cell parameters such as cell shape, size, and membrane properties.^[^ ^161^ ^]^ A device example that uses DEP to separate, trap, and analyze single cells was published by Wu et al.^[^ ^42^ ^]^ and was discussed in Section 2.1.1. In this work, crossover frequency was used to separate single cells. By adjusting the frequency applied to an array of actuation electrodes and medium conductivity, cells could be deflected and steered into the desired outlets. A bipolar electrode array was then used to trap, rotate, and propel single cells. Rotation rate as a function of frequency is often used in combination with modeling and fitting algorithms to extract dielectric properties of cells. In a different study performed by Elitas et al. DEP was used to trap single *Mycobacterium smegmatis* cells and characterize membrane altering mechanisms related to cell death, drug tolerance, and drug resistance using crossover frequencies.^[^ ^162^ ^]^ Single‐cell suspension of bacterial cells was introduced in a low conductivity buffer and two low‐frequency signals were used to center the cells along the midline of the 20 μm wide channel. A varying high‐frequency signal was applied to induce a translational movement of the live cells relative to the midline of the channel. The displacement values were used to calculate the Clausius–Mossotti factors for live cells, which provide a measure of cell polarizability compared to its surrounding medium (Table S1, Supporting Information). Fluorescence microscopy was concurrently used to visualize the process and validate the results.

#### 3.2.3 Electrophoretic Cell Analysis in Integrated Microfluidics

Electrophoresis refers to the movement of charged particles in a uniform electric field guided by electrostatic, i.e., Coulomb, forces that are proportional to the particle's charge. This form of separation can take place in different media, such as hydrogels that act as a sieving matrix (gel electrophoresis), or in capillaries filled with electrolyte solutions (capillary electrophoresis).^[^ ^163^ ^]^ As cells are usually considered electrically neutral, they cannot be easily moved using electrophoresis. Therefore, electrophoresis is not commonly used to manipulate cells, but to sort other charged particles such as water‐in‐oil droplets or biomolecules such as DNA, RNA, or proteins.^[^ ^164^ ^]^ An integrated microsystem‐based application of electrophoretic analysis will be discussed in Section 4.1.1.^[^ ^165^ ^]^ An example for capillary electrophoresis on‐chip was demonstrated by Huang et al. where the designed integrated microfluidic system was able to capture, lyse, label, separate, and quantify the protein contents of single cells.^[^ ^166^ ^]^ In this work, single cells were captured in a reaction chamber using microvalves, where they were lysed and their lysates were labeled with fluorescently labeled antibodies. Target molecules (here, proteins of a low copy number, i.e., less than 1000 molecules cell^−1^) were separated from excess reagents electrophoretically, and were counted at the end of the electrophoretic separation channel by monitoring the number of fluorescence bursts generated.

### 3.3 Mechanical Analysis of Cells in Integrated Microfluidics

The mechanical properties of cells are intrinsic biophysical markers that can be used for the assessment of cell state and health, which can aid in the diagnosis of certain diseases.^[^ ^167^ ^]^ For example, the stiffness of heart cells dramatically changes when a person develops arrhythmogenic right ventricular cardiomyopathy (ARVC). This can be observed already in the early stages or just before ARVC disease, which makes cell stiffness a criterion that could be used for early diagnosis.^[^ ^168^ ^]^ Another such example are cancer cells, which differ from healthy cells in terms of their elasticity.^[^ ^169^, ^170^ ^]^ Due to the high clinical relevance of cellular mechanical properties, the development of novel methods that can analyze the mechanical signatures of individual cells in a low‐cost and high‐throughput manner has become an active area of research in microanalytics. Mechanical characteristics of individual cells, quantified in parameters such as stiffness, viscoelasticity, or deformability, have been characterized by applying various mechanical forces onto them, such as shear stress, compression, or stretching. So far, numerous microfluidic‐based techniques have been developed to provide mechanical deformation of cells on‐chip, including micropipette aspiration or the application of optical tweezers for stretching or compression.^[^ ^171^, ^172^, ^173^, ^174^ ^]^ These methods commonly required user input (such as the micropipette aspiration of individual cells) and were therefore limited in throughput. The importance of automated systems to overcome this challenge was already exemplified by Gossett et al. in 2012, where the authors demonstrated the use of a microfluidic platform to inertially focus, hydrodynamically stretch, image, and analyze single cells in flow.^[^ ^175^ ^]^ This automated microfluidic system was capable of probing single‐cell deformability at approximately 2000 cells s^−1^ by delivering single cells to a stretching extensional flow region where they were deformed at high strain rates, imaged with a high‐speed camera, and computationally analyzed to extract quantitative parameters. Here, the deformability of native populations of leukocytes and malignant cells in pleural effusions could be rapidly assayed and the disease state in patients with cancer and immune activation was predicted with a sensitivity of 91% and a specificity of 86%. While hydrodynamic stretching for cellular mechanical analysis still remains a popular method, alternative stretching methods are commonly explored, such as stretching based on magnetic tweezers or DEP.^[^ ^176^, ^177^, ^178^, ^179^ ^]^ Furthermore, acoustophoresis has emerged as a powerful and versatile tool that can help enable high‐throughput mechanical analyses.^[^ ^180^ ^]^ Morales et al. effectively merged acoustic wave techniques to manipulate cells and optical interferometry within a microfluidic channel.^[^ ^181^ ^]^ The innovative design of this platform featured a piezoelectric transducer used to focus and deform cells acoustically, alongside a laser‐based interferometer to detect changes in the cells’ optical patterns. This integration allowed for the detailed examination of various physical aspects of cells, such as their shape, flexibility, size, and optical properties like refractive index.

**Figure 7:**
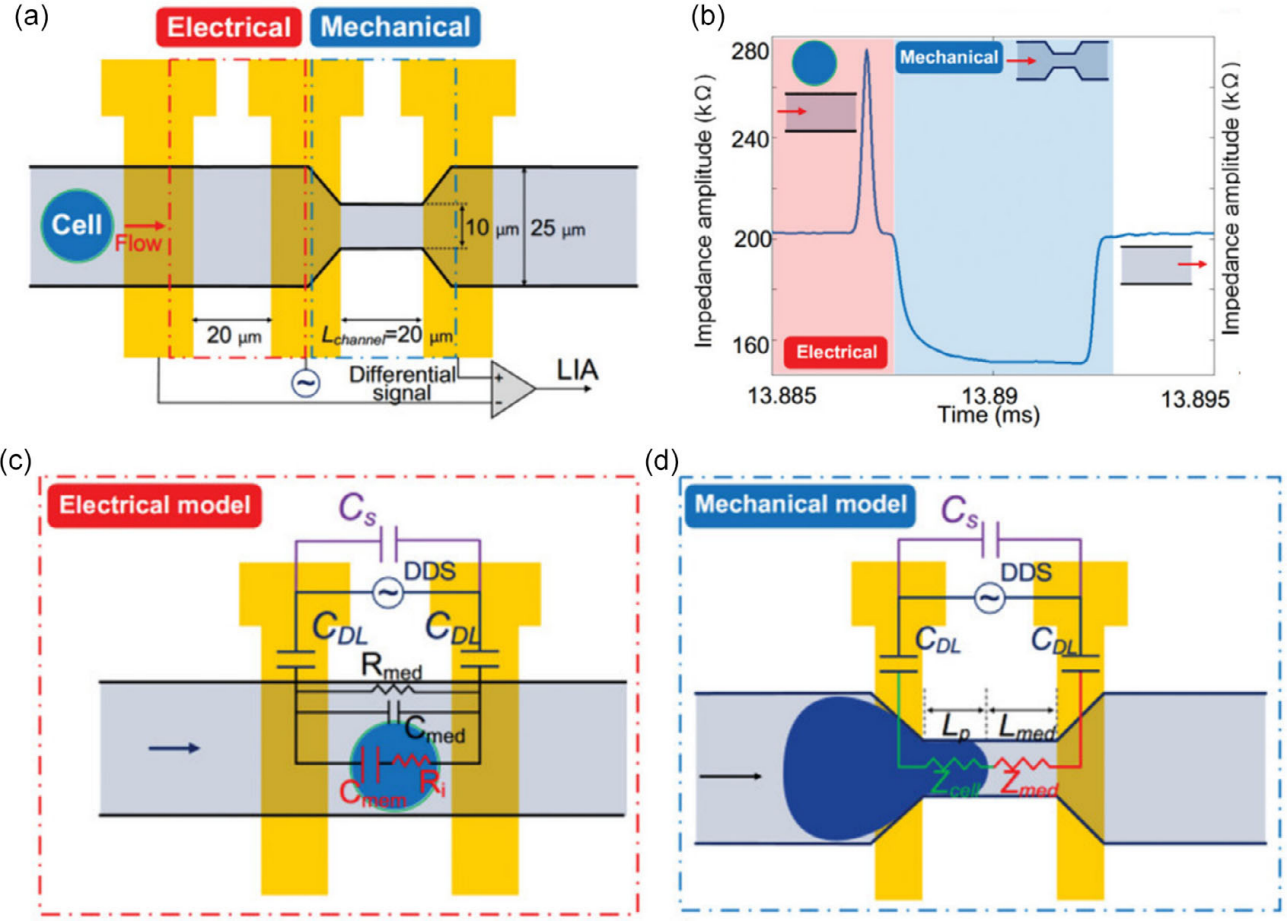
Simultaneous electrical and mechanical characterization of single cells via impedance measurements. a) Illustration of the differential electrode configuration placed at the entrance of a constriction channel. b) As single cells flow through the microfluidic channel, their impedance is measured at the electrical and mechanical sensing areas, yielding different impedance signals. c,d) Obtained impedance signals can be analyzed in accordance with the corresponding equivalent electrical and mechanical models, revealing intrinsic cellular properties without image processing steps. For the electrical model, the impedance (*Z*) consists of solution resistance (*R*_med_), solution capacitance (*C*_med_), cytoplasmic resistance (*R*_*i*_), cell membrane capacitance (*C*_mem_), electric double‐layer capacitance (*C*_DL_), and stray capacitance (*C*_s_). In the mechanical model, *L*_p_ and *L*_med_ represent the length of cell protrusion and medium in the constriction channel, respectively. Adapted with permission.^[^ ^182^ ^]^ Copyright 2023, Wiley.

Recent systems not only focus on higher throughputs and automated data analysis, but also the analysis of various cellular properties at once through multiple modes or methods (i.e., multimodal analyses). A recent multimodal analysis platform was reported by Feng et al. who introduced an impedance‐based electrical–mechanical flow cytometry framework for the label‐free characterization of single cells.^[^ ^182^ ^]^ In this work, the authors aligned differential electrodes at the inlet of a constriction channel to establish an electrical and a mechanical sensing area (**Figure** **7**). Cells passing through these consecutive sensing areas caused a differential impedance signal, which could be fed into corresponding electrical and mechanical models to obtain five intrinsic cell parameters: radius, cytoplasm conductivity, specific membrane capacitance, Young's modulus, and fluidity. Using these parameters, the authors could differentiate between three different cancer types or identify changes in the cytoskeletal structures of cells due to pharmacological treatments. Notably, the operation of this system required no image processing for mechanical property assessment of cells. Such advancements in analysis methods can lead to higher levels of miniaturization and integration, especially for applications that take place outside of laboratory settings.

## 4 Recent Advances in Integrated Single‐Cell Separation and On‐Chip Analysis

In the previous sections, prominent cell separation and analysis methods were introduced and discussed. Later, we discuss recent integrated applications that make use of multiple methods simultaneously to enable integrated single‐cell isolation and on‐chip analysis. We broadly group the discussed microfluidic platforms based on their application domains, namely, platforms used for the analysis of somatic cells (with an emphasis on cancer cells and immune cells), stem cells, and microorganisms. In our discussion, we focus on highlighting the broad integrated device design considerations specific to each application domain, however, an overlap is expected for multiple biological investigations. A compact overview of the integrated systems discussed in this manuscript can be found in **Table** **2**.

**Table 2:** A simplified overview of the integrated microsystems presented in this manuscript

Platform aim	Off‐chip sample preprocessing	On‐chip workflows		
		Cell/particle sorting	Single‐cell/particle isolation	On‐chip analysis
**Analysis of cancer cells**				
Label‐free separation and on‐chip culture of single CTCs^[^ ^188^ ^]^	CTCs were spiked into human peripheral blood obtained from healthy donors in varying concentrations (down to 1000 cells mL^−1^)	Shear‐induced diffusion	Micropillar array	Biological analysis (morphology, proliferation)
				Optical analysis
Dynamic monitoring of CTC metabolites^[^ ^189^ ^]^	CTCs were spiked into human peripheral blood obtained from healthy donors in varying concentrations (<1000 cells mL^−1^)	Inertial focusing Microtraps	Microtraps and microchambers	Biological analysis (metabolomics) Optical analysis
On‐chip single‐CTC immunoblotting^[^ ^165^ ^]^	CTCs were suspended in red‐blood‐cell‐depleted clinical blood samples	Inertial focusing Filtration	Microwells	Biochemical analysis (proteomics) Electrical analysis (electrophoresis) Optical analysis
Enhanced CTC capture^[^ ^56^ ^]^	CTCs mixed with nontarget cells were suspended in cell culture medium	Magnetophoresis	Magnetophoresis	Biological analysis (morphology)
		Hydrodynamic focusing		Optical analysis
On‐chip DNA marker amplification of cancer cells^[^ ^190^ ^]^	Viable human leukemia cells were washed with deionized water prior to their injection into the chip	–	Optical tweezers	Biochemical analysis (genomics)
			Microwells	Optical analysis
On‐chip nucleic acid quantification via LAMP^[^ ^191^ ^]^	Human leukemia cells were suspended in DEP buffer	–	DEP (traditional + insulator‐based) Microtraps and microchambers	Biochemical analysis (genomics) Optical analysis
On‐chip quantification of enzymatic activity^[^ ^132^ ^]^	Breast adenocarcinoma cells were suspended in DEP buffer	–	DEP	Biochemical analysis (proteomics)
			Microtraps and microchambers	Optical analysis
On‐chip ramanome analysis^[^ ^192^ ^]^	Human bladder, lung, renal, and breast cancer cells were suspended in DEP buffer (additional cell types: yeast, microalgae, bacteria)	–	DEP	Biological analysis (metabolomics)
			DLD	Optical analysis
Intracellular delivery of molecules to single cells^[^ ^193^ ^]^	Human melanoma cells were suspended in cell culture medium (additional cell types: human bronchial epithelium and human cardiac fibroblasts)	–	Microwells (vacuum assisted)	Biological analysis (transcriptomic, drug response)
				Optical analysis
Investigation of different drug concentrations on single cells^[^ ^194^ ^]^	Human hepatoma and breast cancer cells were suspended in cell culture medium	–	Microtraps	Biological analysis (drug response)
				Optical analysis
**Analysis of immune cells and immune interactions**				
Label‐free immunophenotyping of leukocytes^[^ ^199^ ^]^	Whole blood samples obtained from healthy donors were lysed or diluted; for glucose‐treated samples, whole blood samples were treated with glucose before lysis	Inertial focusing	Inertial focusing	Biological analysis (Morphology)
				Electrical analysis (EIS)
Enzymatic secretion profiling of leukocytes^[^ ^200^ ^]^	Undiluted whole blood—no sample preparation	DLD	Droplet microfluidics	Biological analysis (proteomics)
				Optical analysis
Migration profiling of dendritic cells^[^ ^196^ ^]^	Human PBMC‐derived dendritic cells were suspended in human serum	–	Microtraps	Biological analysis (morphology, migration)
				Optical analysis
Cytokine secretion analysis under chemical stimulation^[^ ^195^ ^]^	Human monocytes were purified from peripheral blood and suspended in cell culture medium	–	Microtraps (microvalve assisted)	Biological analysis (proteomics)
				Optical analysis
Codetection of multiple secreted factors in relation to cell proximity and migration behavior^[^ ^202^ ^]^	Human oral squamous carcinoma cells and human primary oral squamous carcinoma cells were suspended in cell culture medium; human primary CAF cells were isolated from patient samples and suspended in cell culture medium, human primary monocytes were isolated from PBMCs and differentiated into macrophages	–	Microchambers	Biological analysis (morphology, migration)
				Optical analysis
Fusion of bone‐derived dendritic cells and colon carcinoma cells^[^ ^203^ ^]^	Bone‐marrow‐derived dendritic cells and colon carcinoma cells were suspended in DEP buffer	–	DEP (traditional DEP + insulator based)	Total cell analysis (cell fusion, morphology)
			Microwells	Optical analysis
Real‐time monitoring of cellular interactions^[^ ^204^ ^]^	Natural killer cells and leukemia cells were suspended in cell culture medium		Droplet microfluidics	Total cell analysis (viability, cellular interactions)
			Microtraps (microvalve assisted)	
				Optical analysis
**Analysis of other somatic cells**				
Monitoring neuronal growth^[^ ^6^ ^]^	Cortical neurons were dissociated from E18 embryos of Sprague‐Dawley rats and suspended in cell culture medium	–	DEP	Biological analysis (morphology)
				Optical analysis
Mechanical characterization of HUVECs after their on‐chip culture under varying stimuli^[^ ^208^ ^]^	HUVECs were suspended in cell culture medium	–	Chemical detachment	Biological analysis (morphology, stiffness)
			DEP	Optical analysis
			Microtraps	Mechanical analysis
**Analysis of stem cells**				
Monitoring osteogenic differentiation^[^ ^213^ ^]^	Single‐bone‐marrow mesenchymal stem cells were suspended in cell culture medium	–	Droplet microfluidics	Biological analysis (impedance, membrane capacitance)
				Electrical analysis (EIS)
Monitoring migration behavior of stem cells subjected to chemical stimuli^[^ ^214^ ^]^	Human cardiac stem cells derived from left atrial appendages were suspended in cell culture medium	–	Microchambers	Biological analysis (morphology, proliferation, migration)
				Optical analysis
**Analysis of microorganisms**				
Monitoring the motility of magnetotactic bacteria^[^ ^222^ ^]^	*Magnetospirillum gryphiswaldense* were cultured in growth medium	–	Microchambers	Biological analysis (motility)
				Optical analysis
Investigation of photosynthetic activity of algae^[^ ^223^ ^]^	*Chlamydomonas reinhardtii* were cultured in growth medium	–	Microtraps Microchambers	Biological analysis (proliferation, photosynthetic activity)
				Optical analysis
Investigation of oxygen availability on bacterial growth^[^ ^267^ ^]^	*Escherichia coli* were cultured in growth medium	–	Microchambers	Biological analysis (viability, proliferation)
				Optical analysis
Monitoring antibiotic susceptibility^[^ ^224^ ^]^	*Escherichia coli* and *Salmonella typhimurium* were cultured in growth medium	–	Microtraps	Biological analysis (motility, drug response)
				Optical analysis
Identification and antibiotic susceptibility testing of single bacterial cells^[^ ^225^ ^]^	Clinical urine samples were filtered and diluted	–	Droplet microfluidics	Biochemical analysis (transcriptomic, drug response)
				Optical analysis

### 4.1 Integrated Platforms for the Separation and Analysis of Somatic Cells

#### 4.1.1 Cancer Cells

Traditional bulk analyses often miss the heterogeneity of cancer cells, which plays a significant role in understanding tumor dynamics, drug resistance, and metastatic potential.^[^ ^114^, ^132^ ^]^ To explore tumor dynamics on integrated microfluidic systems, researchers typically use cells sourced from cancer cell lines, spike cancer cells into human whole blood samples to mimic CTCs in a liquid biopsy (i.e., spiking experiments), or use clinical liquid biopsy samples.

The analysis of CTCs presents distinct challenges due to their scarcity in the bloodstream (estimated 1–10 CTCs mL^−1^ of blood) and the lack of specific markers indicative of metastasis‐promoting cells.^[^ ^114^, ^183^, ^184^ ^]^ Furthermore, CTCs may exist as clusters rather than single cells, complicating their handling in microfluidic devices.^[^ ^185^, ^186^ ^]^ Lastly, the expectation for direct isolation and analysis of CTCs from blood samples without prior off‐chip processing adds a layer of complexity to the design of integrated microfluidic platforms. Despite such challenges, systems capable of executing complete workflows from whole blood samples have been emerging.^[^ ^187^ ^]^ For example, Zhou et al. reported a micropillar array that can trap CTCs sorted from untreated whole blood for long‐term on‐chip analyses.^[^ ^188^ ^]^ Here, human hepatoma (HepG2) cells were used as a model and spiked into human peripheral blood samples in varying concentrations (down to 1000 cells mL^−1^). Sorting was realized by shear‐induced diffusion in a sandwich flow configuration (i.e., the phosphate‐buffered saline [PBS], buffer flow was “sandwiched” by blood flow). This configuration prevented RBC‐induced clogging and allowed size‐based sorting as larger cells (here, >15 μm, such as HepG2) migrated into the buffer flow and moved on to the trapping area to be captured at the micropillar array. With the addition of a growth medium, HepG2 cells could be cultured on‐chip for up to 5 days, allowing for migration and proliferation studies, which were performed optically using immunofluorescent stains.

**Figure 8:**
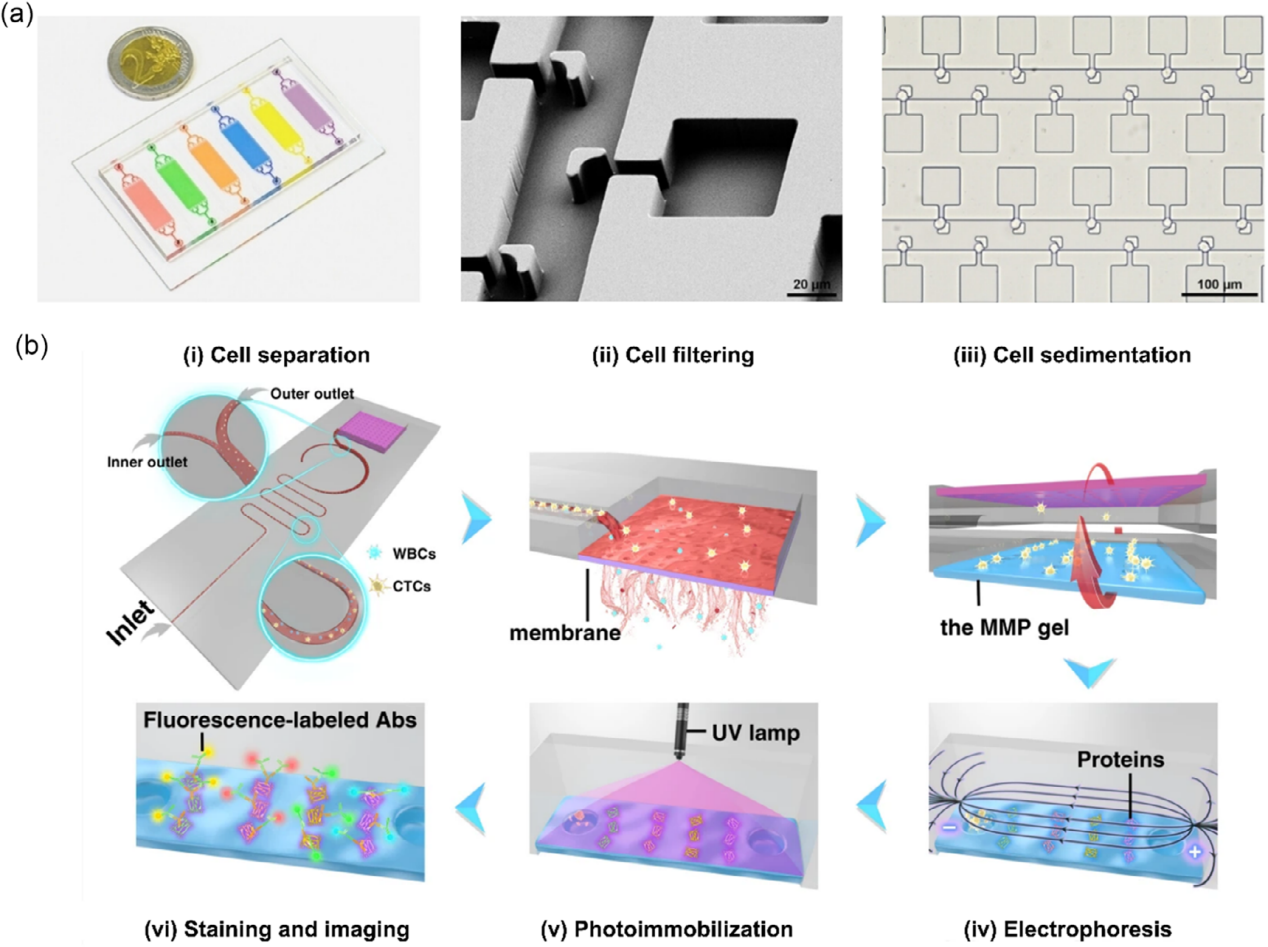
a) SingMAC platform used for single‐cell profiling of extracellular lactate and intracellular glucose. Photograph of six parallel microfluidic chips (left panel), scanning electron microscope image of the hook‐shaped traps and their adjacent microchambers (middle panel), and microscope image of captured MCF‐7 cells (right panel). Adapted with permission.^[^ ^189^ ^]^ Copyright 2023, Wiley. b) Schematic illustration of the integrated microfluidic single‐cell immunoblotting chip: i) Cell separation in zigzag microchannels. CTCs are isolated from background white cells in RBC‐depleted samples; ii) CTC purification and enrichment using an integrated membrane; iii) gravity‐induced settling of CTCs into microwells on the polyacrylamide gel upon chip inversion; iv) chemical lysis of CTCs and the separation of released proteins via electrophoresis; v) in‐gel UV immobilization of separated proteins; vi) fluorescence staining and confocal imaging of the released proteins to acquire proteomic data on‐chip. Adapted under the terms of CC BY 4.0 license.^[^ ^165^ ^]^ Copyright 2022, The Authors. Published by Springer Nature.

Similarly, a simple yet highly advanced platform reported by Gao et al. (termed “SingMAC”) could identify malignant CTCs spiked into unprocessed whole blood at concentrations below 1000 cells mL^−1^.^[^ ^189^ ^]^ This platform was designed for the dynamic monitoring of tumor cell metabolites at a single‐cell level, specifically extracellular lactate and intracellular glucose. It employed a wheel‐shaped radial inlet structure used to separate cell clusters to prevent clogging along with a dense array of hydrodynamic hook‐shaped traps used to isolate single CTCs of 10–20 μm size (**Figure** **8**a). RBCs were observed the escape the microtraps due to their size and biconcave disk shape, while some large WBCs were trapped in the hooks. Yet, even at relatively low spike concentrations, a cancer‐cell capture efficiency of 80% was achieved. Traps captured the CTCs in a “squashed” state, therefore causing a mechanical stress resembling the physical conditions experienced by CTCs during extravasation. Metabolite monitoring took place in microchambers paired to each microtrap, which were filled with a reaction buffer containing enzyme‐loaded metal‐organic frameworks (MOFs). The catalysis of the CTC extracellular lactate and intracellular glucose by the enzymes embedded in MOFs yielded a fluorescent signal revealing semiquantitative metabolic information. By using this information, the authors were able to identify spiked breast cancer cells (MCF‐7 and triple‐negative MD Anderson‐Metastatic Breast‐231 [MDA‐MB‐231]) showing high lactate and glucose levels in whole blood for five different blood samples obtained from healthy patients. Here, the authors noted that despite their high tumorigenic capacity, MDA‐MB‐231 cells do not possess the specific biomarkers required for detection through conventional immune‐based techniques, such as the CellSearch assay, the established standard for CTC detection.^[^ ^189^ ^]^

Abdulla et al. recently reported an integrated system for the label‐free isolation, enrichment, and on‐chip immunoblotting of single CTCs derived from a breast‐cancer‐positive patient (Figure 8b).^[^ ^165^ ^]^ Here, the obtained blood samples were initially lysed (i.e., depleted of RBCs) and the nucleated cells were resuspended in a saline solution. On‐chip, patient‐derived CTCs were separated from WBCs in a zigzag and semicircular channel influenced by inertial lift, Dean drag, and centrifugal forces. Subsequently, further purification using an integrated membrane filter significantly increased the CTC enrichment factor. Enriched CTCs were collected in microwells made of an immunoblotting‐compatible polyacrylamide hydrogel by flipping the chip, and chemically lysed by the introduction of a lysis buffer. Lysates from individual CTCs underwent on‐chip electrophoresis for separation, followed by UV irradiation to immobilize these proteins in the hydrogel. This setup enabled on‐chip fluorescence labeling and confocal microscopy analysis to study the protein expression profiles of single CTCs, outperforming bulk statistical analysis for epithelial cell adhesion molecule (EpCAM, a cancer‐related antigen) detection and profiling.^[^ ^114^ ^]^ Furthermore, it demonstrated the sophisticated integration of multiple techniques and materials (e.g., filtration membrane, immunoblotting hydrogel) into integrated devices.

To address common limitations of cell isolation using magnetic fields, such as low capture efficiency due to inadequate magnetic force in microfluidic systems, Seyfoori at al. suggested the use of smart, anti‐EpCAM antibody‐conjugated magnetic microgels.^[^ ^56^ ^]^ In this work, a soft micromagnet pattern was integrated into a microfluidic channel and used to localize an external magnetic field to generate a high magnetic field gradient (see Section 2.1.2). This device design enabled high capture efficiency and purity of targeted, magnetic microgel‐tagged MCF‐7 cells suspended in a Dulbecco's Modified Eagle Medium (DMEM) with nontarget lymphoblastic leukemia cells (Jurkat). Furthermore, magnetic microgels were designed in a thermoresponsive manner, releasing a fluorescent dye molecule when heated to 37 °C. The release of the fluorescent dye into the trapped cells allowed for immediate cell staining, facilitating the on‐chip monitoring of cells without additional operation steps.

Unlike the fully integrated analysis of CTCs, which necessitates initial sorting of CTCs from whole blood, the integrated analysis of cancer cells derived from cell lines does not demand intricate on‐chip cell sorting mechanisms. This simplifies overall device design, allowing for the exploration of nonconventional strategies for on‐chip manipulation and analysis. For example, Cong et al. employed optical tweezers in a microfluidic device with gold‐coated PDMS microwells to trap single human leukemia cancer cells.^[^ ^190^ ^]^ In this setup, the laser served a dual purpose: trapping single cells and inducing localized thermal effects for extended manipulation, such as cell transport. This effect was achieved by stimulating the gold layer on the microwells, which led to a temperature rise at the laser beam's focal point, subsequently causing fluidic convection within the microwells. The authors harnessed this optothermal phenomenon alongside optical tweezing to precisely guide single cells into designated 3D microwells. Additionally, they utilized the heat generated by this process to locally lyse living human leukemia cells, facilitating the release of nucleic acids for in situ isothermal DNA marker amplification. Yet, the method was limited in throughput due to inherent operation principles of optical tweezers, as discussed in Section 2.1.4.

**Figure 9:**
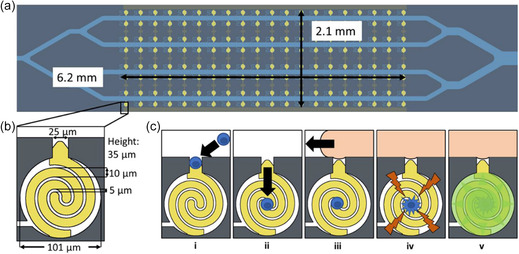
Illustration of the integrated microfluidic device used to screen the enzymatic activity of cancer cells. a) The analysis area consisting of 160 individual analysis chambers interfaced with a bipolar electrode array. b,c) Illustration of the interlocking spiral electrode design facilitating trapping and lysis of captured cells, independent of their location in the chamber. Adapted with permission.^[^ ^132^ ^]^ Copyright 2023, American Chemical Society.

For higher‐throughput analyses, different cell separation and lysis methods (e.g., chemical, electrical) are explored. For example, Qin et al. suggested a DEP‐assisted self‐digitization chip, which makes use of traditional and insulator‐based DEP to guide cell/particle movement (see Section 2.1.1).^[^ ^191^ ^]^ In this work, in situ nucleic acid quantification via loop‐mediated isothermal amplification (LAMP) was performed once single human leukemia cells (K562) were trapped in individual microchambers via DEP forces. A similar DEP and microarchitecture combination was shown by Banovetz et al. who reported a bipolar electrode‐based DEP platform overlaid by an array of microchambers to capture, isolate, lyse, and analyze single MDA‐MB‐231 cells on‐chip (**Figure** **9**).^[^ ^132^ ^]^ Here, the designed bipolar electrodes not only facilitated cell trapping, but also cell lysis. Particularly, the interlocking spiral electrode design ensured the successful lysis of the trapped single cells independent of their location in the chamber. This platform was used to analyze enzymatic activity in situ, in contrast to a genomic analysis discussed in the previous example.^[^ ^191^ ^]^ Finally, Wang et al. introduced a novel integrated platform combining DEP with a DLD array for focusing and trapping fast moving cells in a wide channel to allow for their downstream full‐spectrum spontaneous single‐cell Raman analysis.^[^ ^192^ ^]^ This platform could analyze multiple cell types, including yeast, microalgae, bacteria, and human cancers (human bladder [T24], lung [A549], renal [OSRC‐2], breast [MCF‐7] cancer cell lines) to produce high‐throughput, heterogeneity‐resolved ramanomes.

Integrated microsystems have also been utilized for intracellular deliveries and drug studies. Dong et al. used a silicon, pyramid‐shaped micropore array in combination with on‐chip electroporation to enable intracellular delivery of small (≈22 bp) and macro molecules (≈9 kb) to single human melanoma cells (A375).^[^ ^193^ ^]^ In this device, single cells were held in place in the microtraps using controlled vacuum and a focused electric field was used to electroporate more than 100 000 cells in parallel. Following electroporation, the molecules of interest (oligonucleotides, plasmid DNA, chemotherapeutic drugs) could be introduced into the permeabilized cells. Cells continued to be cultured in their traps for post‐transfection monitoring (e.g., viability testing after intracellular drug delivery and in situ mRNA detection) via microscopy. The integrated nature of this microsystem allowed better uniformity, higher delivery efficiency and better cell viability in comparison to a commercial electroporation system. Furthermore, the system could be utilized for studying noncancerous cell lines, including human bronchial epithelium bronchial epithelium transformed with Ad12‐SV40 2B (BEAS‐2B) and human cardiac fibroblasts. Shen et al. recently reported a multi‐concentration microfluidic gradient generator combined with a single‐cell capture array to study the effects of varying drug concentrations on HepG2 and MCF‐7 cells.^[^ ^194^ ^]^ This innovative platform featured a Tai Chi‐spiral mixer to generate 24 distinct concentration gradients from a single drug or two drugs. In addition to mixing and gradient generation, this system isolated single cells based on their size and deformability using a specially designed capture array. This allowed for an unprecedented level of analysis, examining how individual cells with different physical properties react to various drug concentrations. With this integrated platform, the authors observed that smaller and/or more deformable tumor cells demonstrated greater resistance to the drugs, compared to their larger and/or less deformable counterparts. Additionally, the combined use of 5‐fluorouracil and cisplatin was found to be more effective in inhibiting the growth of HepG2 cells compared to individual drug treatments. The integration of multi‐concentration gradient generation with single‐cell capture based on biomechanical characteristics represents a significant advancement in the field of drug screening and personalized medicine, offering insights into optimizing chemotherapy regimens for individual cellular responses.

#### 4.1.2 Immune Cells and Immune Interactions

Characterizing single immune cells and their interactions plays a crucial role in understanding immune mechanisms and developing new tools and strategies for various applications including vaccine development, cancer immunotherapy, and drug discovery. Immune cells can be sourced from cell lines (e.g., natural killer cells), tissue (e.g., bone marrow), or liquid biopsies (e.g., cerebrospinal fluid, blood). Furthermore, they can be generated (e.g., dendritic cell generation and maturation from peripheral blood mononuclear cells [PBMCs]), or mimicked (e.g., establishing antibody secretion via transfection of cell lines).^[^ ^195^, ^196^, ^197^, ^198^ ^]^

**Figure 10:**
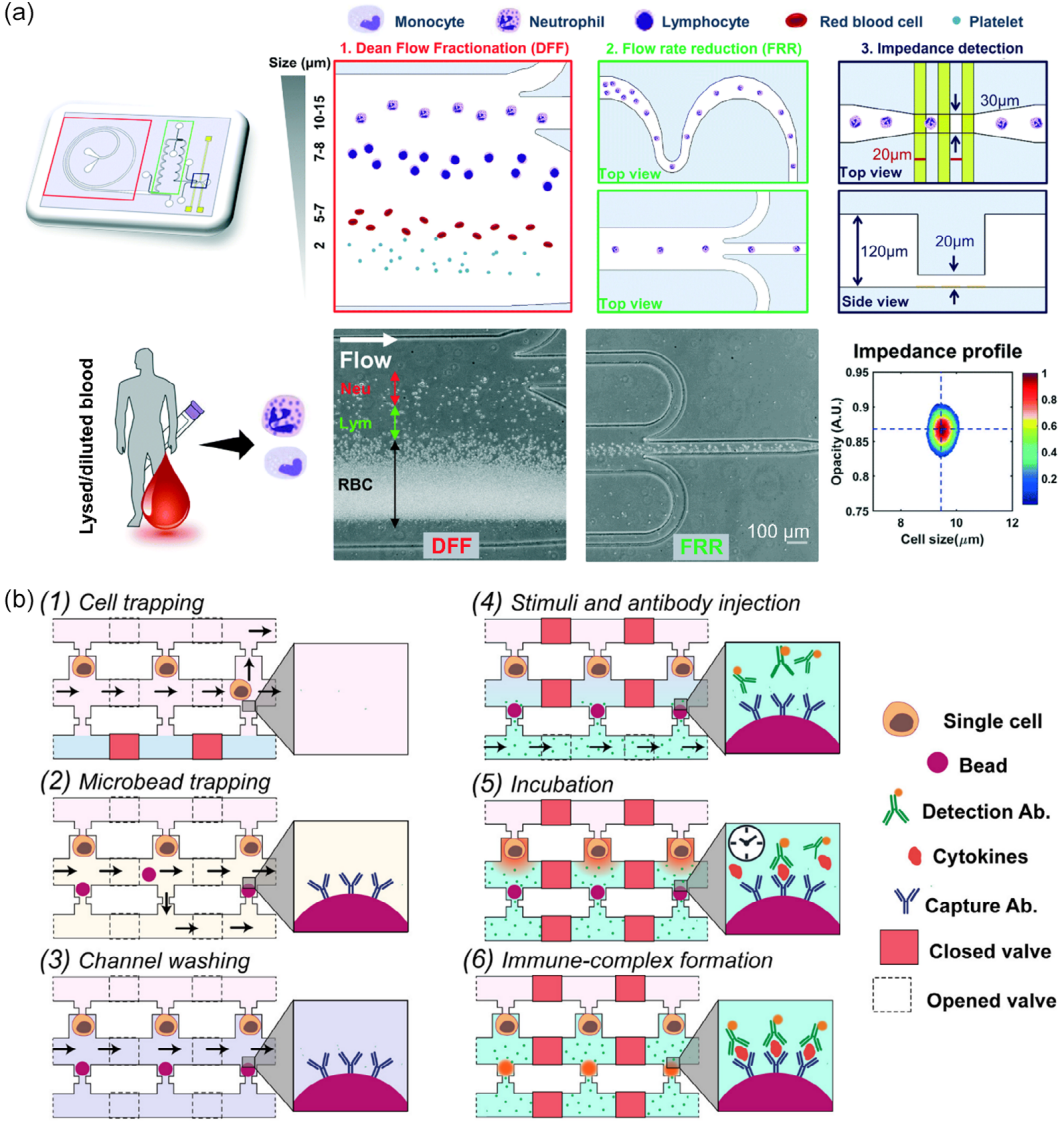
a) Rapid and label‐free sorting and electrical phenotyping of neutrophils and monocytes. DFF enables initial sorting of neutrophils and monocytes from diluted or lysed blood samples (panel outlined by red square). DFF‐purified neutrophils and monocytes are inertially focused into a single stream in serpentine channels (panel outlined by green square) prior to impedance detection (panel outlined by black square). Adapted with permission.^[^ ^199^ ^]^ Copyright 2019, Royal Society of Chemistry. b) Illustration of the assay workflow designed for the secretion profiling of single monocytes. 1 and 2) Single cells and functionalized beads are captured in their respective traps via a series of valve operations. 3) Upon co‐trapping, channels are flushed with fresh media. 4) Microvalves on the central channels and cell‐side channels are closed to deliver chemical stimuli (to induce cell secretion) and detection antibodies into each chamber. 5) With the closure of all microvalves, potential cross talk between individual chambers is prevented. Cells are incubated for 24 h. 6) Formation of immunocomplexes is observed via fluorescence microscopy. Adapted with permission.^[^ ^195^ ^]^ Copyright 2023, American Chemical Society.

In contrast to CTCs, WBCs (i.e., leukocytes, such as neutrophils, lymphocytes, monocytes) are relatively abundant in blood (estimated 4.5–11.0 × 10^6^ mL^−1^). This facilitates their direct capture and isolation from whole blood samples for revealing insights into the pathogenesis of various diseases and the identification of new prognostic biomarkers. For example, Petchakup et al. proposed single neutrophil impedance signatures as a potential biomarker for diabetes.^[^ ^199^ ^]^ In this work, the authors developed an integrated microfluidic system for the rapid (under 5 min) and label‐free separation and electrical characterization of leukocytes from diluted or lysed whole blood samples. Using Dean flow fractionation (DFF) in spiral channels, neutrophils and monocytes were size‐separated and then analyzed for electrical impedance post flow rate reduction (**Figure** **10**a). Importantly, the on‐chip separation of leukocytes from RBCs and platelets prior to electrical characterization allowed error‐free impedance readouts. The authors used this system to analyze the formation of neutrophil extracellular traps (NETosis) in neutrophils, a mechanism associated with combating pathogens, and showed that the dielectric properties (size and opacity) of healthy versus glucose‐treated neutrophils were significantly different due to varying NETosis formation. Similarly, Zeming et al. demonstrated a series of microfluidic chips connected by tubing to process whole blood samples (50 μL) and profile multiplexed enzymatic secretion of single leukocytes in 60 min.^[^ ^200^ ^]^ This system executed six processes: leukocyte sorting, cell washing, fluorescent enzyme substrate mixing, droplet generation, incubation, and real‐time readout. For leukocyte sorting, a DLD array with L‐shaped micropillars was employed, which achieved 99.7% purity and a 700‐fold enrichment. This array also facilitated cell preparation by navigating leukocytes to assay media, eliminating traditional washing and resuspension steps. The sorted leukocytes were encapsulated in microfluidic droplets with enzymatic substrates, and their enzyme profiles were analyzed using an integrated photomultiplier tube across four fluorescent channels. Using this platform to analyze samples for patients suffering from acute heart failure, the authors revealed patient‐specific immune responses and a reduction in immune‐cell enzymatic secretion levels as the patients recovered.

Integrated microfluidic systems can also help study the behavior of single immune cells to elucidate poorly characterized cellular actions. Shao et al. trapped single dendritic cells derived from PBMCs in a microtrap array with orthogonal migration channels leading to open reservoirs. This setup imitated a lymph vessel environment, facilitating dendritic cells bidirectional migration in response to various chemoattractants.^[^ ^196^ ^]^ In another study by Cedillo‐Alcantar et al. single monocytes and antibody‐functionalized beads were co‐trapped in 500 pL microchambers to analyze cytokine secretion under different chemical stimuli for 24 h (Figure 10b).^[^ ^195^ ^]^ A key feature of this microsystem was the integrated microvalves. They ensured the isolation of cell–bead pairs in individual chambers, preventing cross‐contamination and inter‐chamber communication. Furthermore, they enabled on‐demand solution exchange, playing a critical role in the immunoassay (e.g., by loading of the detection antibodies) and the delivery of varying (concentrations) stimuli in different chip sections. While not demonstrated in this study, the authors also suggested the use of the on‐demand solution exchange mechanism for the maintenance of single‐cell cultures over extended periods. This work can also be used to highlight a common challenge when immune interactions are to be studied, namely, efficient trapping and pairing of multiple cell types, such as immune and cancer cells or bacteria, to create single‐cell co‐cultures.^[^ ^201^ ^]^ Microfluidic systems commonly employ Poisson distribution for cell pairing, leading to a high incidence of non‐pairing events (see the discussion on cell–droplet pairing in Section 2.2.4). For example, Li et al. developed an innovative antibody barcode‐based chip to profile tumor‐stromal and tumor‐immune secretions in relation to cellular proximity and migration behavior.^[^ ^202^ ^]^ This system was designed with a high‐density microchamber array where single cells were confined and tracked. On top of the microchambers was a glass slide patterned with spatially resolved antibodies, allowing for the codetection of various secreted factors such as proteins and extracellular vesicles. Despite its potential to yield high information content, only about 6% (≈600) of the microchambers achieved single‐cell cocultures due to the limitations of Poisson distribution‐based pairing. To allow for more precise trapping and pairing in their system, Cedillo–Alcantar, as briefly discussed earlier, made use of hydrodynamic traps combined with a series of microvalve operations.^[^ ^195^ ^]^ This method allowed the authors to reach cell occupancy rates of up to 94% and single‐cell‐bead pairing efficiencies of 56%, though this included multi‐bead pairings. In another study, an insulator‐based DEP chip was used for pairing and fusing bone‐marrow‐derived dendritic cells with colon adenocarcinoma cells Colon Tumor 26 (CT26) in microwells, optimizing various parameters such as microwell diameter and depth, flow rate, and electric field strength.^[^ ^203^ ^]^ The authors reported cell‐trapping efficiencies of up to 86% for CT26 and 80% for dendritic cells. Their method allowed for an approximate cell–cell pairing efficiency of ≈80% and the electrofusion of paired cells with fusion efficiencies up to 70%. Fusion of dendritic and tumor cells is of high interest in cancer immunotherapy applications due to its potential use in cancer vaccinations.

In addition to pairing single cells in microchambers, droplets can be used as a pairing medium. Agnihotri et al. encapsulated natural killer cells (NK92) and leukemia cells (K562) within droplets, each approximately 1.8–4.2 pL in volume.^[^ ^204^ ^]^ Although the study did not specify the pairing efficiency, it effectively utilized these droplets for the real‐time monitoring of the interactions between killer and tumor cells. The droplets were trapped at docking sites downstream using hydrodynamic resistance, and the cytotoxic activity of killer cells was observed via fluorescence imaging. A notable feature of the system was the selective release of droplets containing cells of interest (such as fast killers) by actuating microvalves aligned on top of the droplet docking sites. Microvalve actuation altered the hydrodynamic resistance at specific docking sites, enabling targeted retrieval of cells for further analyses.

#### 4.1.3 Other Somatic Cells

As highlighted by the numerous examples discussed in Section 4.1.1 and 4.1.2, most published works on single‐cell isolation and analysis in integrated microfluidic systems focus on studying single cancer and immune cells. This focus is driven by the urgent need to understand and treat diseases, especially cancer. While the single‐cell analysis of other somatic cells (e.g., neurons or endothelial cells) can also provide an essential biological tool for understanding fundamental physiological processes and tissue‐specific functions, establishing these studies on integrated microsystems can be limited by technical challenges.^[^ ^205^ ^]^

**Figure 11:**
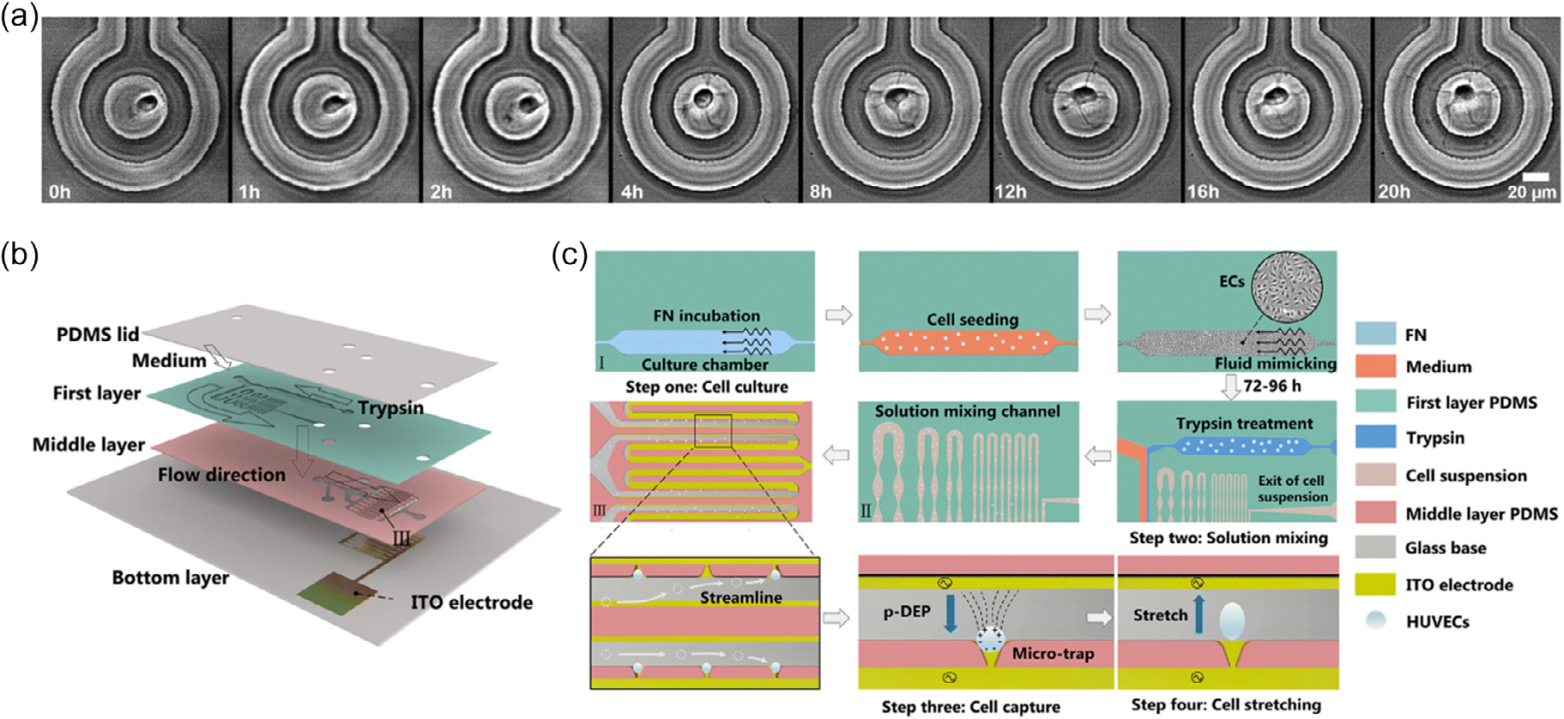
a) Integrated microfluidic platform designed to isolate, trap, and culture single neurons at specified positions using dielectrophoretic trapping. Time‐lapse phase contrast images of a living neuron are shown. Neurons could be cultured on‐chip for over a week. Adapted under the terms of CC BY 4.0 license.^[^ ^6^ ^]^ Copyright 2018, The Authors. Published by Springer Nature. b) Design and c) operational workflow of the multilayer‐integrated microfluidic platform enabling long‐term on‐chip culture and mechanical analysis of endothelial cells. The platform consists of two distinct modules, one for the dynamic cell culture and the other for mechanical property assessment via DEP‐based trapping and stretching. Cultured cells are subjected to varying shear stresses or chemicals and consecutively detached via trypsin treatment. Detached single cells are captured downstream for mechanical analysis. Adapted with permission.^[^ ^208^ ^]^ Copyright 2023, Royal Society of Chemistry.

Most somatic cells, in contrast to CTCs and WBCs, need to adhere to a solid surface to remain viable and proliferate.^[^ ^136^, ^138^ ^]^ This commonly requires the tailoring of the integrated microfluidic systems to allow for cell attachment (e.g., ECM protein coating), long‐term on‐chip cell culture, as well as the detachment of the cells for downstream single‐cell analyses. Nevertheless, cell attachment is most often not desired on all chip surfaces as nonspecific cell adhesion might prevent, for example, targeted cell isolation. Some examples we discussed in Section 4.1.1 have presented ways to overcome this problem. For instance, the micropillar array presented by Zhou et al. captured spiked HepG2 cells and allowed for their on‐chip culture for up to five days.^[^ ^188^ ^]^ The authors did not pre‐coat the chip with an ECM protein, hence not allowing for the initial cell attachment onto the pillars. Instead, following the isolation of cells, they introduced a growth medium to the chip that included fetal bovine serum. Fetal bovine serum includes plasma proteins that may adsorb to the micropillar array, hence promoting the attachment of the pre‐isolated cells.^[^ ^206^, ^207^ ^]^ In contrast, Shen et al. compartmentalized the cell culture and gradient generator sections of the integrated microfluidic system so that the cell culture sections could be treated with fibronectin independent of the rest of the platform. Similar strategies have been developed to culture neurons or endothelial cells on integrated microfluidic platforms. For example, Kim et al. used DEP to isolate and trap single neurons at distinct positions to monitor their growth (**Figure** **11**a).^[^ ^6^ ^]^ This device was coated with poly‐D‐lysine prior to cell loading to ensure cell attachment to the chip. Single neurons were trapped at the center of individual ring‐shaped electrodes by energizing the electrode closest to them. When an electrode was occupied by a single neuron, other cells were repelled using negative dielectrophoretic forces. By maintaining constant voltage, the authors were able to replace cell culture media without affecting the distinct positions of single neurons prior to establishing neuron attachment. Once the cells were aligned at their desired positions, the applied voltage was removed and neurons were allowed to settle. Settled neurons could be cultured on‐chip for over a week and their growth was tracked via real‐time live‐cell imaging.

Yang et al. recently developed a sophisticated, highly integrated multilayer microfluidic chip to analyze the mechanical properties of human umbilical vein endothelial cells (HUVECs) under conditions mimicking the human vascular environment.^[^ ^208^ ^]^ The chip consisted of distinct modules for dynamic cell culture and mechanical property assessment using DEP‐based trapping and stretching. To enable cell attachment, the cell culture area was initially coated with fibronectin. HUVECs adhered to the culture area and were cultivated for 4 days under various flow‐induced shear stresses. Post culture, cells were detached using trypsin treatment for 2 min and were then navigated through a solution mixing channel toward the analysis area. Challenges like cell clumping were mitigated by using an hourglass‐shaped channel design that enhanced convective diffusion, effectively dispersing cell aggregates and preventing channel clogging. In the downstream analysis area, the authors utilized pDEP for cell capture, wherein single cells, influenced by the electric field gradient, were directed into microtraps. This process yielded a capture efficiency of ≈79%. Once trapped, cells were subjected to DEP‐induced stretching using an AC signal, enabling the measurement of single‐cell mechanical properties like Young's modulus (Figure 11b). The study revealed that increased shear stress led to a higher Young's modulus in HUVECs, indicating a stiffening of cells. Conversely, the application of tumor necrosis factor alpha (TNF‐α, an inflammation reducer) and blebbistatin (a cytoskeleton disruptor) demonstrated a decrease in cell stiffness. This innovative platform not only enabled a 4 days of in vivo–mimicking cell culture, but also allowed for precise measurements of single‐cell mechanics. Such integrated platforms can replace labor‐intensive manual measurement methods, such as atomic force microscopy, and provide valuable insights for cardiovascular disease research, drug testing, and understanding biomechanical properties in a physiologically relevant setting.

### 4.2 Integrated Platforms for the Analysis of Stem Cells

Stem cells, known for their remarkable self‐renewal capacity and ability to differentiate into multiple lineages, present a unique biological complexity.^[^ ^209^ ^]^ While stem cells are commonly considered as somatic cells, they can be classified in different categories depending on their differentiation potential and source type (e.g., adult stem cells, embryonic stem cells, induced pluripotent stem cells) and therefore are discussed here under a separate section.^[^ ^210^, ^211^, ^212^ ^]^ Through single‐cell analysis, researchers can delve into the subtle variations and evolving conditions within what appear to be uniform stem cell populations, unraveling the intricate regulatory networks and molecular mechanisms that underpin stem cell pluripotency and their commitment to specific lineages. Additionally, single‐cell analysis can be instrumental in identifying rare subsets and transient states of stem cells, thereby enriching the understanding of stem cell biology. The insights gained from such in‐depth analysis are likely to enhance the development of advanced stem‐cell‐based therapies, deepen the understanding of embryonic development, and boost the fields of tissue engineering and regenerative medicine.

Despite the potential of single‐stem‐cell analysis, designing integrated microfluidic systems that can perform complete stem‐cell workflows on‐chip (e.g., long‐term culture, selective subjection to differentiation medium and/or stimuli for defined periods, subsequent on‐chip analysis) remain challenging. These challenges are similar to those we have discussed in Section 4.1.3 and will not be discussed here further. However, systems that demonstrate the significance of integrated single‐stem‐cell analysis have been emerging. Fan et al. developed a droplet‐based microfluidic device integrated with a microelectrode array for the impedance analysis of single‐bone‐marrow mesenchymal stem cells at different levels of osteogenic differentiation.^[^ ^213^ ^]^ In this system, bone marrow mesenchymal stem cells were initially cultured in an osteogenic differentiation medium off‐chip. Cells at varying stages of cell differentiation (days 0, 7, 14, and 21) and across different cell passages (6, 7, and 11) were then introduced to the chip and were encapsulated in microdroplets generated at a T junction. Individual droplets were trapped downstream, aforementioned integrated electrodes using a bypass channel geometry. By carrying out impedance measurements, the authors revealed notable cellular heterogeneity among bone marrow mesenchymal stem cells during osteogenic differentiation. Höving et al. used a microchamber‐based trapping method to subject stem cells to varying stimuli on‐chip. In this work, the authors studied the migration patterns and proliferation behavior of single primary adult human cardiac stem cells cultured in microchambers when exposed to human blood plasma.^[^ ^214^ ^]^ This integrated microsystem allowed for the on‐chip culture and real‐time tracking of single stem cells for up to 48 h, revealing human blood plasma as a significant chemical stimulus contributing to the faster migration of human cardiac stem cells. Stem cell migration toward specific locations in the body is associated with processes such as the regeneration of injured tissues.

### 4.3 Integrated Platforms for Single‐Microorganism Analysis

The analysis of single microorganisms such as bacteria, fungi, or algae can help researchers better understand fundamental biological processes, decode microbial–environmental interactions, or identify new characteristics that can be further employed for new biotechnological applications. Due to the broadness of this research field and the large diversity and complexity of microorganisms, different aspects of how microfluidic technologies can be used for the analysis of single microorganisms have been previously reviewed.^[^ ^16^, ^215^, ^216^, ^217^, ^218^, ^219^, ^220^, ^221^ ^]^ In this section, we provide representative recent examples of integrated microfluidic devices designed for the isolation and on‐chip analysis of single microorganisms. Similar to many examples discussed in the previous sections, single microorganisms are often trapped in microtraps/wells/chambers and are subjected to a stimulus, then analyzed via chosen means. For example, Codutti et al. conducted a study on the motility of individual magnetotactic bacteria (*Magnetospirillum gryphiswaldense*), examining their behavior under an external magnetic field.^[^ ^222^ ^]^ This approach aimed to replicate the bacteria's natural habitat, offering a more realistic environment compared to standard laboratory conditions. Szeles et al. studied the photosynthetic activity of *Chlamydomonas reinhardtii*, a single‐celled algae, over several hours.^[^ ^223^ ^]^ Kasahara et al. established oxygen‐controlled microfluidic cultures for *Escherichia coli* to study the effects of oxygen availability on microbial growth, morphological properties, and physiological processes.

**Figure 12:**
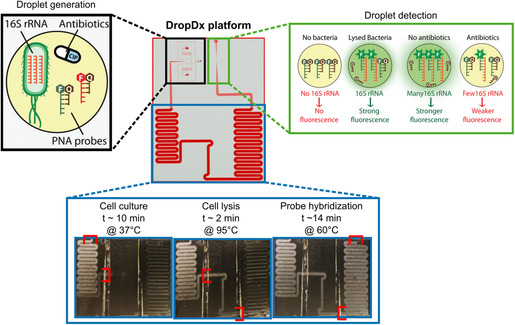
Droplet‐based integrated microfluidic device (DropDx platform) for the identification and antimicrobial susceptibility testing of pathogens found in clinical urine samples in case of a urinary tract infection. The device initially encapsulated single bacteria in droplets together with an antibiotic and a fluorogenic hybridization probe (here, peptide nucleic acid [PNA] probe) targeting their ribosomal RNA (16S rRNA), shown in the black panel. Single bacteria in droplets were cultured for approximately 10 min to expose them to the antibiotics, and then were lysed, and lysates were labeled by fluorogenic probes, shown in the blue panel. As each process step required a different temperature, the chip was fixed onto a temperature rig with three heating zones via thermally conductive paste. The fluorescent signal of the individual droplets was detected at the droplet detection region, where the color and intensity of the signal revealed the pathogen type and the antibiotic susceptibility, respectively, shown in the green panel. Adapted under the terms of CC BY 4.0 license.^[^ ^225^ ^]^ Copyright 2021, The Authors. Published by Wiley.

One application field that has gained significant traction in recent years is the analysis of single microorganisms for screening drug susceptibilities. This is especially relevant for bacterial populations, which are showing increasing resistance to broad‐spectrum antibiotics due to their common and nontargeted use. Pitruzzello et al. employed an integrated microfluidic platform equipped with microtraps to isolate and monitor the motility of single microbial cells, including various strains of *Escherichia coli* and *Salmonella typhimurium*.^[^ ^224^ ^]^ The authors exposed these bacteria to different concentrations of antibiotics both in the integrated platform and via a broth microdilution assay, and established a strong correlation between drug‐induced inhibition of motility and growth. By employing single‐cell motility as a measure for antibiotic susceptibility, the authors revealed the heterogenic response of single bacteria to antibiotics, which is not possible with traditional antibiotic susceptibility testing (AST) that measures population‐wide averages. Furthermore, it was possible to establish a minimum inhibitory concentration in just 1.5 h, significantly faster than conventional growth‐based methods (16 h). Integrated microfluidic devices that can combine AST with rapid pathogen detection might prove to be valuable tools both in clinical and point‐of‐care settings. For example, the integrated, droplet‐based microfluidic platform designed by Kaushik et al. “DropDx”, could identify single pathogens in filtered and diluted clinical samples of urine and perform AST in 30 min to provide rapid information related to urinary tract infections.^[^ ^225^ ^]^ The authors achieved this pace by using fluorogenic hybridization probes to detect 16S rRNA, a component of the bacterial ribosome. In contrast to other nucleic acid sequences (e.g., DNA) commonly used for molecular pathogen identification, 16S rRNA is abundant (approximately 10^3^–10^5^ copies cell^−1^), therefore allowing for the identification of bacteria without the need for nucleic acid amplification. In addition, the amount of 16S rRNA served as a surrogate for bacterial growth and changes in its quantity under antibiotic exposure provided a basis for AST. To realize rapid detection and AST on‐chip, single bacteria were encapsulated in droplets containing hybridization probes for pathogen identification as well as an antibiotic for AST. The droplets were cultured on‐chip for about 10 min, followed by bacterial lysis in a designated area. The released 16S rRNA was then targeted by fluorogenic probes, generating a fluorescence signal whose color and intensity revealed the pathogen type and the results of the AST (**Figure** **12**). The authors demonstrated the potential clinical utility of this platform by testing 50 patients’ urine samples and acquiring results agreeing with the standard clinical methods (i.e., plating and culturing urine samples for 8 h to assess the pathogenic load, mass spectroscopy analysis for pathogen identification and broth microdilution requiring up to 48 h for AST).

## 5 Discussion and Future Perspectives

### 5.1 The Rise of Integrated Microfluidic Platforms

The development of microfluidics marked a pivotal shift in analytical capabilities. The power of miniaturization, stemming from the interplay of physical forces at the microscale, facilitated the development of systems capable of conducting rapid, highly sensitive, and precise analyses, while concurrently minimizing the consumption of samples and reagents. This era notably aligns with the vision of conducting total analyses on a single platform, as initially proposed by Manz et al. through their hallmark publication that introduced the concept of a “miniaturized total (chemical) analysis system (μTAS)”.^[^ ^226^ ^]^ In the same period, the terms “lab‐on‐chip (LOC)” and “integrated microfluidics” emerged in scholarly literature, often used in an interchangeable manner. While LOC was usually defined by the integration of one or multiple laboratory functions in a single microsystem, integrated microfluidics referred to the incorporation of various microscale components and functionalities (optical, fluidic, electronic, mechanical) on a single platform to perform complex tasks.^[^ ^227^, ^228^, ^229^, ^230^, ^231^, ^232^ ^]^ Following the introduction of PDMS and soft‐lithography techniques by Whitesides, microsystems technologies witnessed a substantial increase in accessibility, which catalyzed a broad scientific endeavor toward actualizing the concepts of μTAS, LOC, and integrated microfluidics.^[^ ^233^ ^]^ Already in 2004, Erickson and Li published a review paper discussing advances in integrated microfluidic devices, with applications spanning from on‐chip DNA analysis to cell handling.^[^ ^232^ ^]^

### 5.2 Technical Challenges to Full Integration and Proposed Solution Strategies

While the terms μTAS, LOC, and integrated microfluidics have been extensively used in the past two decades, true integration can only be achieved when single microfabricated platforms can accommodate full workflows that might contain several complex steps. In the context of single‐cell analysis, the development of such complex microsystems that can host complete single‐cell workflows is far from simple as it relies on the characteristics of the cells to be analyzed (e.g., adherent or nonadherent cells), the combination of methods required for cell separation and analysis, as well as the type of analysis required (e.g., biological, biochemical, or both). From a technical perspective, combining several cell separation and analysis methods that often rely on a variety of working principles and require contrasting device design criteria is challenging. For example, microsystems made out of glass are most commonly sealed using thermal bonding, which requires that systems are heated to a few hundred degrees Celsius. When other elements are integrated into these systems, e.g., microoptical elements made of photoresist, process compatibility needs to be evaluated to ensure high quality of features.

One way to overcome such challenges, especially when required methods or processes are in some way incompatible, is to further adopt plug‐and‐play configurations, as discussed in two examples earlier.^[^ ^69^, ^148^ ^]^ Briefly, Antfolk et al. bonded an acoustophoretic chip facilitating rare cell pre‐concentration and a DEP chip to trap rare cells in an array of electroactive microwells for further analysis.^[^ ^69^ ^]^ The use of separate but connected chips allowed the authors to overcome limitations regarding the integration of bulk piezoelectric transducers into the microsystem. Similarly, Debnath et al. connected a blood‐plasma‐separation chip to a commercial SPR sensor chip via microfluidic tubing, which eliminated the need for complex sensor surface preparation steps.^[^ ^148^ ^]^ Although such plug‐and‐play configurations are not by definition one integrated system, they might allow for integrated workflows performed in overall integrated platforms. In one of the examples, we have discussed in Section 4.1.2, enzymatic secretion of leukocytes found in the whole blood samples of patients suffering from acute heart failure were profiled, revealing patient‐specific immune responses. Similar to the two examples discussed earlier, this system consisted of separate microfluidic chips connected via microfluidic tubing. The interesting approach here was the use of tubing as a droplet incubation line, making it a functional part of the platform.^[^ ^200^ ^]^ This further demonstrates that integration can come in many forms and the definition of an integrated platform can take many shapes. However, while plug‐and‐play configurations address certain technical integration challenges by providing more space and separation, they commonly require manual assembly/operation and are prone to contamination. For these reasons, monolithic platforms are still the standard.

The production of compartmentalized monolithic devices that can accommodate multiple workflows is no easy feat. The task becomes even more complex when integration of actuation or sensing elements such as microoptical elements is necessary for cell isolation or analysis. As a result, highly integrated monolithic devices primarily rely on passive cell sorting and isolation methods that do not require complex fabrication, while maintaining respectable accuracy, versatility, and compact operation. In fact, the majority of the integrated microfluidic systems discussed in this manuscript employ passive cell separation techniques for cell isolation prior to single‐cell analysis (Table 2). A notable exception is DEP, which is widely employed both as a stand‐alone or as a supporting technology for sorting, capturing, relocating, fusing, or lysing single cells. The widespread use of DEP in comparison to other active methods can be attributed to its easy integration into microdevices and the high throughput it allows. This is in contrast to other active cell isolation and analysis methods, such as optical tweezers, that allow highly precise manipulations at the cost of expensive integration and low throughput. In a similar fashion, there are integrated MEMS platforms that do not use microfluidics for precise single‐cell isolation and analysis, yet they find limited applications. An example is MEMS microgrippers, which have been developed for single‐cell isolation and analysis based on a variety of actuation principles.^[^ ^234^ ^]^ Such devices are significantly limited in throughput and are complex to fabricate and operate, which limits their broad adoption for integrated operations.

### 5.3 Limitations of Affinity‐Based Separation and Advances in Label‐Free Operation

A further aspect to be considered when multiple workflows are integrated into a single device is that downstream processes can be affected by the choice of methods used upstream. For example, while affinity‐based cell separation commonly offers high selectivity and specificity that result in high subpopulation purity, cell capturing makes retrieving cells for downstream analysis difficult. Moreover, affinity‐based separation methods that rely of cell capturing often inherently alter the physical characteristics of the cell surface, which might negatively affect further cell analysis steps. The use of affinity‐based capturing is particularly noteworthy to discuss in the context of CTCs. Most microfluidic systems that aim to capture and isolate CTCs employ anti‐EpCAM conjugation, a method also demonstrated by Seyfoori et al. in Section 4.1.1.^[^ ^56^ ^]^ In this work, the authors labeled MCF‐7 cells with anti‐EpCAM antibody‐conjugated magnetic microgels for their effective capture using a magnetic field. However, MCF‐7 cells, as discussed by the authors, are known to abundantly express EpCAM, which is not the case for all CTCs.^[^ ^56^, ^114^ ^]^ Moreover, EpCAM‐based labeling might not efficiently capture CTCs that are undergoing epithelial‐to‐mesenchymal transition, which is a key step in the metastatic cascade resulting in the loss of the epithelial phenotype of the cancer cells and therefore reducing EpCAM expression.^[^ ^114^ ^]^

To overcome such limitations of label‐based CTC capturing, multiple label‐free systems, commonly relying on passive separation methods, have been developed. Here, we have discussed one particular example, demonstrated by Gao et al. where the authors trapped CTCs in microtraps and evaluated their tumorigenic ability by evaluating their expression of extracellular lactate and intracellular glucose.^[^ ^189^ ^]^ Such integrated systems may pave the way for a more comprehensive understanding of cancer cells, leading to advanced detection and analysis methods without the need for labels.

### 5.4 Integrated Microfluidic Platforms for Clinical Applications

Despite the demonstrated possibility of label‐free operation, the effectiveness of most integrated platforms aiming to analyze CTCs from clinical whole blood samples remains uncertain. This is due to the fact that various examples we have discussed in Section 4.1.1 have demonstrated the capability of their platforms by spiking cancer cells into whole blood samples obtained from healthy patients at approximately 1000 cells mL^−1^.^[^ ^188^, ^189^ ^]^ While eliminating the need for blood preprocessing is a significant advancement for integrated microsystems, CTC concentrations in clinical samples are typically estimated to range from 1 to 10 cells mL^−1^, which implies the need for off‐chip CTC enrichment.^[^ ^183^ ^]^ We were only able to identify one recent platform that demonstrated the isolation and analysis of CTCs from clinical blood samples, however, its operation also required off‐chip preprocessing including lysis, centrifugation, and resuspension of the nucleated cells, thereby significantly enriching the CTC population.^[^ ^165^ ^]^

Clinical samples of urine (which are neither as viscous as blood nor pose a risk of coagulation) were analyzed in a different publication.^[^ ^225^ ^]^ Preprocessing in the form of filtering was performed to deprive them of particulates which pose a risk of channel clogging and to increase the occurrence of droplets with no bacteria but high fluorescence intensities. In addition, urine samples needed to be diluted, as they caused a high autofluorescence background that interfered with the intended fluorescence measurement. While this platform showed results that are in agreement with standard clinical laboratory methods, samples with an estimated pathogenic load less than 30 000 CFU mL^−1^ were not analyzed. These examples point to the further advancements needed for the complete on‐chip processing of patient‐derived liquid biopsies and the need for developing systems sensitive enough to process a low number of target cells.

Beyond liquid biopsies, microsystems that can process patient‐derived tissue biopsies are also being developed. Recently, multiple microfluidic systems that can perform on‐chip tissue dissociation have been reported, a process traditionally requiring multiple manual steps.^[^ ^235^, ^236^, ^237^ ^]^ Although to date an integrated system that allows single‐cell analysis starting with a tissue biopsy has not been demonstrated, we envision that on‐chip tissue dissociation will eventually be merged with established on‐chip cell separation and analysis to enable complete workflows in an integrated manner.

### 5.5 Integrated Microfluidic Platforms for Point‐of‐Care Operations

The use of integrated microsystems for single‐cell analysis in clinical settings can also be extended to the point of care. Notable examples from our literature review include the use of neutrophil impedance signatures as a diabetes biomarker^[^ ^199^ ^]^ and the identification of single pathogens in urine for the diagnosis of urinary tract infections.^[^ ^225^ ^]^ The integrated nature of similar platforms, combined with the portability inherent to miniaturization and the possibility for result read‐out using smart phones makes these systems attractive for use in remote areas or at home. However, significant challenges remain in terms of cost and scalability, including the miniaturization of often bulky peripheral instrumentation. Some of these challenges could be overcome by the use of cheaper materials, such as plastics or paper, as well as the use of fabrication techniques that can be easily scaled, such as injection molding instead of soft lithography. In line with these strategies, various systems are under development. For instance, a paper‐based platform that utilizes screen printed electrodes and capillary flow for DEP‐based plasma separation was explored.^[^ ^238^ ^]^ Recently, Faraghat et al. fabricated a DEP‐based microfluidic platform by structuring conductor–insulator laminates via a desktop cutting plotter.^[^ ^239^ ^]^ This system could be used for the high‐throughput continuous separation of yeast cells in a low‐cost manner, without the need for wet lab facilities for the production of the platforms.

### 5.6 Defining “Integration” in a Broader Manner

In our review and discussions so far, we have only considered examples of integrated systems that follow a traditional single‐cell analysis route, namely, cell sorting, followed by single‐cell isolation and analysis. However, an inverse route is also possible. Cell sorting and isolation could also be the outcome or purpose of cell analysis. Some examples include fluorescence‐activated, image‐activated, and Raman‐activated cell sorting and isolation. Such systems initially analyze cells and sort them into specified outlets based on their properties (e.g., the localization of fluorescence proteins). However, the operation of these systems may be limited by the necessary use of labels or established markers. For example, if the elasticity of a cell population has not been thoroughly characterized, it would not be possible to use elasticity as a marker to sort and isolate cells.

Similarly, throughout this review, we have particularly focused on integrated microfluidic devices that can perform on‐chip analysis. Nevertheless, today there are platforms available that can perform multiple on‐chip functions/process steps related to sample preparation needed for off‐chip analysis of single cells. One prominent application field is the analysis of single‐cell genomes and transcriptomes via benchtop sequencers. Microfluidics systems have been used to perform a series of operational steps such as the capture, isolation, lysis, and sequence amplification of single cells, however, genomic/transcriptomic analysis is performed off‐chip.^[^ ^240^, ^241^, ^242^ ^]^ Another example are microfluidic platforms that enable single‐cell isolation and preparation (e.g., lysis) for off‐chip mass spectrometry.^[^ ^243^, ^244^, ^245^ ^]^ Considering the complex nature of such analyses, it could be argued that such platforms should still be classified as integrated given their multifunctional nature and ability to perform complex single‐cell workflows, despite the absence of on‐chip single‐cell analysis.

A similar argument could be made for platforms that utilize microscopy and/or machine or deep learning algorithms for single‐cell classification. The complexity and abundance of microscopes and computers makes the need for their miniaturization obsolete. Such microsystems would deviate from the classic “sample‐in‐answer‐out” definition of integrated microsystems, which raises the question whether complete integration and miniaturization should always be the goal. Herein, we have defined integrated microsystems as systems that can perform complete single‐cell workflows on chip. While we particularly focused on systems that also demonstrate integrated on‐chip analysis, we believe the definition of a complete workflow should not necessarily include an analysis step. Whereas distinctions can be made for truly integrated systems that abide by the classic “sample‐in‐answer‐out” definition, we believe a broader or looser definition is more appropriate.

### 5.7 Future Perspectives

In summary, fully integrated microsystems have the potential to revolutionize single‐cell analysis by upholding common advantages of microfluidics while eliminating the need for off‐chip sample processing. Throughout this manuscript, we have discussed multiple emerging examples capable of isolation and analysis of different types of single cells to obtain content‐rich information through the integration of various workflows on a single platform. We believe the recent advances in microfabrication methods, especially ones that allow rapid prototyping, in combination with ever increasing throughput and sinking costs could allow integrated microsystems to become the next generation platforms used to decipher cellular heterogeneities. Increasing accessibility of the platforms, combined with efforts to enable multi‐modal analyses and quick data acquisition and processing,^[^ ^182^, ^246^ ^]^ could pave the way toward new biological pathways, leading to the better understanding of various (patho)physiological processes. Furthermore, integrated systems can be used for molecular and cellular diagnostics, making them not only a valuable tool for research environments, but also for clinical settings and eventually even at the point of care.

## Conflict of Interest

The authors declare no conflict of interest.

## Author Contributions

All authors contributed to literature review, writing and editing the manuscript; H.K. did the visualization; M.V. and I.C. supervised the project.

## Supporting information

Supplementary Material
