# Supplementary material for: Integrated Microfluidics for Single‐Cell Separation and On‐Chip Analysis: Novel Applications and Recent Advances
Source: Small Sci. 2024 Feb 2;4(4):2300206. doi: 10.1002/smsc.202300206 (PMC11935165; doi:10.1002/smsc.202300206)
Supplement: Supplementary file 1 — Supplementary Material [file SMSC-4-2300206-s001.pdf]

Integrated microfluidics for single-cell separation and on-chip analysis: novel applications and recent advances

Hazal Kutluk, Martina Viefhues, Iordania Constantinou

H.K., I.C.:

Institute of Microtechnology (IMT), Technische Universität Braunschweig, Alte Salzdahlumer Str. 203, 38124 Braunschweig, Germany

Center of Pharmaceutical Engineering (PVZ), Technische Universität Braunschweig, Franz-Liszt-Str. 35a, 38106 Braunschweig, Germany

M.V.:

Experimental Biophysics & Applied Nanoscience, Faculty of Physics, Bielefeld University, Universitätsstrasse 25, 33615 Bielefeld, Germany

***Corresponding author:** i.constantinou@tu-braunschweig.de

Table S1: Equations governing the theoretical principles behind active cell separation methods.

	Dielectrophoresis (DEP)[1–3]	Magnetophoresis[4–6]	Acoustophoresis[7–10]	Optical tweezers[11,12]
**Governing equations**	$\vec{F}_{DEP} = \alpha\nabla\,\vec{E}^2$<br>$\alpha = 4\,\pi\epsilon_0\epsilon_m R^3\Re(K_{CM})$<br>$K_{CM} = \left(\frac{\epsilon_p^* - \epsilon_m^*}{\epsilon_p^* + 2\epsilon_m^*}\right)$<br>$\mathrm{W} = \sigma\mathrm{E}^2$<br>$\alpha_p > \alpha_m$: positive DEP<br>$\alpha_p < \alpha_m$: negative DEP	$\vec{F}_{mag} = \frac{1}{2}\nabla(\vec{\mu}\cdot\vec{B})$<br>$\vec{F}_{mag} = \frac{\mathrm{V}(\mathrm{X}_p - \mathrm{X}_m)}{\mu_0}(\vec{B}\cdot\nabla)\,\vec{B}$<br>$\mathrm{X}_p > \mathrm{X}_m$: positive magnetophoresis<br>$\mathrm{X}_p < \mathrm{X}_m$: negative magnetophoresis	$F_{ACP} = 4\pi\, a^3 E_{ae} k\sin(2kz)\,\phi$<br>$\phi = \frac{\rho_p + \frac{2}{3}(\rho_p - \rho_m)}{2\rho_p + \rho_m} - \frac{1}{3}\frac{\rho_m c_m^2}{\rho_p c_p^2}$	$F_{Hooke} = -k\Delta x$
**Parameters**	$\vec{F}_{DEP}$: dielectrophoretic force<br>α: relative polarizability of the cell/particle with respect to the medium<br>$\vec{E}$: electric field<br>K_{CM}: Clausius-Mossotti factor<br>$\epsilon_{p,m}^*$: complex dielectric permittivity of the cell/particle and the medium<br>ϵ_0: dielectric permittivity of vacuum<br>R: cell/particle radius<br>**W**: local power generated due to Joule heating<br>σ: conductivity of the electrolyte solution<br>**E**: electric field strength<br>$\alpha_{p,m}$: polarizability of the cell/particle and the medium	$\vec{F}_{mag}$: magnetic force<br>$\vec{\mu}$: magnetic moment of the (dia)magnetic particle<br>$\vec{B}$: magnetic field<br>V: (total) volume of the (dia)magnetic particle(s)<br>μ_0: magnetic permeability of free space<br>$\mathrm{X}_{p,m}$: magnetic susceptibilities of the (dia)magnetic particle and the medium	F_{ACP}: (primary) acoustic radiation force<br>a: cell/particle radius<br>E_{ae}: acoustic energy density<br>k: wavenumber<br>**z:** the distance of the cell/particle from the wall of the microfluidic channel<br>ϕ: acoustic contrast factor<br>$\rho_{p,m}$: density of the cell/particle and the surrounding medium<br>$c_{p,m}$: speed of sound in cell/particle and the surrounding medium	F_{Hooke}: optical trapping force<br>k: virtual spring constant of the cell/particle<br>Δx: deflection of the cell/particle from equilibrium position (i.e., the optical axis of the Gaussian beam)<br>$n_{p,m}$: refractive index of cell/particle and medium
**Assumptions**	Cell/Particle is homogeneous and spherical	Magnetic particle is homogeneous and spherical	Cell/Particle is homogeneous and spherical	Gaussian beam profile; $n_p > n_m$

[1] R. Pethig, *Dielectrophoresis: Theory, methodology, and biological applications*, Wiley, Hoboken, NJ, Chichester, West Sussex **2017**.
[2] J. Regtmeier, R. Eichhorn, M. Viefhues, L. Bogunovic, D. Anselmetti, *Electrophoresis* **2011**, *32*, 2253.
[3] M. Viefhues, R. Eichhorn, *Electrophoresis* **2017**, *38*, 1483.
[4] S. A. Peyman, E. Y. Kwan, O. Margarson, A. Iles, N. Pamme, *Journal of chromatography. A* **2009**, *1216*, 9055.
[5] M. D. Tarn, N. Hirota, A. Iles, N. Pamme, *Science and technology of advanced materials* **2009**, *10*, 14611.
[6] S. S. Leong, Z. Ahmad, S. C. Low, J. Camacho, J. Faraudo, J. Lim, *Langmuir the ACS journal of surfaces and colloids* **2020**, *36*, 8033.
[7] P. Glynne-Jones, R. J. Boltryk, M. Hill, *Lab on a chip* **2012**, *12*, 1417.
[8] R. Barnkob, H. Bruus, ASA **2009**, p. 20001.
[9] S. Karthick, A. K. Sen, *Phys. Rev. Applied* **2018**, *10*, 34037.
[10] K. Olofsson, B. Hammarström, M. Wiklund, *Lab on a chip* **2020**, *20*, 1981.
[11] A. Ashkin, *Proceedings of the National Academy of Sciences of the United States of America* **1997**, *94*, 4853.
[12] T. Yang, F. Bragheri, P. Minzioni, *Micromachines* **2016**, *7*, 90.
